# Neonatal Deletion of *Hand1* and *Hand2* within Murine Cardiac Conduction System Reveals a Novel Role for HAND2 in Rhythm Homeostasis

**DOI:** 10.3390/jcdd9070214

**Published:** 2022-07-04

**Authors:** Rajani M. George, Shuai Guo, Beth A. Firulli, Michael Rubart, Anthony B. Firulli

**Affiliations:** 1 Herman B Wells Center for Pediatric Research, Departments of Pediatrics, Anatomy and Medical and Molecular Genetics, Indiana Medical School, Indianapolis, IN 46202, USA; rmgeorge@iu.edu (R.M.G.); bfirulli@iu.edu (B.A.F.); 2 Division of Cardiology, Department of Medicine, The Krannert Institute of Cardiology, Indiana University School of Medicine, Indianapolis, IN 46202, USA; sg11@iu.edu

**Keywords:** cardiac conduction, HAND factors, electrocardiogram, optical mapping

## Abstract

The cardiac conduction system, a network of specialized cells, is required for the functioning of the heart. The basic helix loop helix factors *Hand1* and *Hand2* are required for cardiac morphogenesis and have been implicated in cardiac conduction system development and maintenance. Here we use embryonic and post-natal specific *Cre* lines to interrogate the role of *Hand1* and *Hand2* in the function of the murine cardiac conduction system. Results demonstrate that loss of HAND1 in the post-natal conduction system does not result in any change in electrocardiogram parameters or within the ventricular conduction system as determined by optical voltage mapping. Deletion of *Hand2* within the post-natal conduction system results in sex-dependent reduction in PR interval duration in these mice, suggesting a novel role for HAND2 in regulating the atrioventricular conduction. Surprisingly, results show that loss of both HAND factors within the post-natal conduction system does not cause any consistent changes in cardiac conduction system function. Deletion of *Hand2* in the embryonic left ventricle results in inconsistent prolongation of PR interval and susceptibility to atrial arrhythmias. Thus, these results suggest a novel role for HAND2 in homeostasis of the murine cardiac conduction system and that HAND1 loss potentially rescues the shortened HAND2 PR phenotype.

## 1. Introduction

The pumping of the mammalian heart is a result of synchronized contractions of atrial and ventricular cardiomyocytes that are coordinated via electrical impulse transmitted through a specialized cell network, referred to as the cardiac conduction system (CCS). Starting in the right atrium, pacemaker cells within the sinoatrial node (SAN), initiate the cardiac electrical impulse [1]. This electrical impulse moves across both atria before its velocity is reduced at the atrioventricular node (AVN), allowing for ventricular diastole. The delayed electrical signal then passes to the bundle of HIS and right and left bundle branches (BBs) releasing their trigger for ventricular cardiomyocyte contraction at the apex of each ventricle through epicardial break through points [2,3]. The electrical signal that triggers the wave of cardiomyocyte contraction through the ventricles is via terminal network of specialized cardiomyocytes termed Purkinje fibers (PF) which compose the ventricular conduction system (VCS) [4]. PF activation and subsequent epicardial breakthrough of the electrical signal results in ventricular systole. Improper development and/or maintenance of any of the CCS components are associated with causation of cardiac arrhythmia and sudden cardiac death [5].

During cardiogenesis, two distinct pools of progenitor cells, the first heart field (FHF) and second heart field (SHF), coalesce at the midline of the developing embryo to form a linear heart tube composed of cardiomyocytes and endocardial cells in contact with the lumen. Initial heart tube contraction initiates at embryonic stage (E) E8.0 in mice and by week 3 gestation in humans [6]. This early cardiac contraction is mediated directly through cardiomyocytes. As the vertebrate heart undergoes cardiac morphogenesis to adopt a four chambered structure, starting at E11.5 in mice, a precise mix of cardiac transcription factors coordinate a subset of cardiomyocytes to specialize into CCS [7]. No transcription factor to date shows complete restriction to cells fated to CCS, as these identified factors play additional roles during cardiogenesis [8,9].

The basic helix loop helix (bHLH) transcription factors HAND1 and HAND2 are expressed in a temporal and tissue-specific pattern during early heart development [10]. Previous work suggests that HAND factors might play a role in the development and perhaps maintenance of CCS. Overexpression of *Hand1* using an inducible *tet-on* system within adult mouse myocardium leads to electrocardiographic changes observed including prolonged PR, QRS, and RR interval, and also shows an increase in expression of *Gja5* [11]. Whole mount in situ analysis demonstrated that deletion of *Hand1* in the developing heart using *Nkx2.5:Cre* results in decreased *Gja5* expression in ventricles at E10.5 [12]. Loss of cardiac *Hand1* and single copy of *Hand2* results in an even more severe reduction of *Gja5* transcript levels [12]. Deletion of *Hand1* using a targeted knockin *Nkx2.5^Cre/+^* allele resulted in aberrant electrocardiogram (ECG) phenotypes with elongated QRS2 duration in Lead I, II, and III, and defects in the VCS, with abnormal epicardial breakthroughs during optical mapping in all mutant hearts assayed [13]. HAND2 has also been implicated in SAN specification using fibroblasts that are induced to pacemaker-like myocytes in cell culture models of cardiogenesis [14]. In these experiments, HAND2 is required to change chromatin accessibility that promotes pacemaker-specific gene expression within fibroblasts undergoing lineage conversion [14].

Recently, HAND1 has been demonstrated to directly play a role in CCS morphogenesis [15]. In a GWAS analysis, two single nucleotide polymorphisms (SNPs) 5′ of *HAND1* were identified in humans who exhibited abnormal QRS intervals [16]. Although the two identifed *HAND1* SNPs are not conserved in mice, their locations flanked an evolutionarily conserved *Hand1* left ventricle (LV) specific enhancer [15]. Deletion of this *Hand1* LV enhancer in mice (*Hand1^ΔLV/^^ΔLV^*) also resulted in pronounced hyperplasia of the VCS as well as a disorganized His bundle. Functionally, these mice presented with abnormal QRS intervals associated with a a right bundle branch block (BBB) [15]. Further interrogation of the enhancer sequence revealed three additional *HAND1* SNPs, one of which altered one of two GATA *cis*-elements necessary for the *Hand1* LV enhancer activity [15]. The resulting mutation of the GATA *cis*-element shows a decreased affinity for GATA4 DNA binding. When this mutation is incorporated into mice (*Hand1^36;75/36;75^*), QRS defects were not observed; however, optical mapping experiments indicated defective VCS function. This suggested that there may be both congenital and homeostatic roles for HAND factors in CCS development and function. To attempt to address this question regarding HAND factor function within the CCS, we utilized the well characterized knock-in mouse *Cre* driver line *Cntn2^3’UTR-IRES-Cre-EGFP^* (*Cntn2^Cre-EGFP/+^*), that expresses *Cre-recombinase* post-natally starting at day 2 to delete *Hand1* and *Hand2* well after CCS development is completed. Given that the available HAND2 CCS function data is in vitro [14], and the finding that LV deletion of *Hand2* contributes to LV cardiomyocyte specification phenotypes [17], we use *Cntn2^Cre-EGFP^* and our *Hand1^LV^-Cre* [17], that expresses *Cre-recombinase* in approximately 90% of LV cardiomyocytes between E8.5–E13.5, to delete *Hand1* and *Hand2* post-natally within the CCS. Our results demonstrate that post-natal loss of HAND1 does not visibly alter ECG parameters. Moreover, optical mapping analysis adult of *Cntn2^Cre-EGF^*; *Hand1^fx/fx^* hearts reveals that the VCS appears to function normally. In contrast, neonatal loss of *Hand2* results in changes in AV conduction with a significantly shortened PR interval. Surprisingly, neonatal CCS deletion of both *Hand1* and *Hand2* does not result in a consistent ECG phenotype, suggesting loss of HAND1 rescues loss of HAND2. Deletion of *Hand2* in the embryonic LV results in inconsistent increases in PR interval at some time points, increased susceptibility to atrial arrhythmias, and increased dyssynchrony between left and right ventricular activation in hearts from male mice. Taken together these findings support the idea that HAND1 function within the CCS appears to be purely congenital, that HAND2 loss alters atrial arrhythmogeneity as well as AV and intraventricular conduction, and that removing HAND1 can restore normal PR interval due to HAND2 loss-of-function.

## 2. Materials and Methods

### 2.1. Mouse Lines and Breeding

Mouse lines *Cntn2^3′UTR-IRES-Cre-EGFP^* (*Cntn2^Cre-EGFP^*) [18], *Hand1^LV^-Cre* [17], *Hand1^tm2Eno^* (*Hand1^fx/fx^*) [12], *Hand2^tm1Cse^* (*Hand2^fx/fx^*) [19] were genotyped by PCR and Southern blotting as previously described [15,20]. Complete list of alleles used for experiments is listed in Table S1 (Supplementary Materials). Mice were randomly assigned numbers at birth and selected for experiments without blinding based on genotype. ARRIVE guidelines 2.0 were used for study design (Table S8). Mice were weaned at 4 weeks and consequently used for ECG analysis between 5 and 25 weeks of age as indicated. All repeated measurements were conducted under standardized conditions.

### 2.2. Surface ECG Recording and Analysis

Male and female mice used in ECG studies were assayed at 5-week, 10-week, 15-week, 20-week, and 25-week time points. The same cohorts of mice for each time point were maintained. Mice were anesthetized with isoflurane administered as 2.5% volume vapor and placed on a heating pad in a supine position with continuous body temperature monitoring using a rectal probe to maintain 37–38 °C. Mice were under anesthesia via mask for 90–120 s and depth of narcosis was assessed by gently pressing on paws and monitoring heart rate. Needle electrodes were placed subcutaneously in the right and left axilla and in the left groin. Bipolar leads I and II signals were simultaneously recorded for a minimum of 1 min using the PowerLab 26T (ADI Instruments, Colorado Springs, CO, USA) with a sampling rate of 2 k/s at 20 mV range. The heart rate and ECG signal from Lead I and Lead II were monitored in real time using the LabChart Pro software (ADI Instruments, Colorado Springs, CO, USA) and recorded.

Analysis of ECG data was carried out using LabChart Pro software (ADI Instruments, Colorado Springs, CO, USA). Lead III data was not analyzed in this study due to inaccuracies identified within the algorithm used to calculate ECG values by the LabChart software. For Lead I and Lead II, ECG signals were averaged by alignment to QRS maximum using a minimum of 100 beats. For analysis, PR interval was measured from the beginning of P-wave (as determined by deflection from isoelectric line) to the beginning of QRS complex. QRS duration was measured from the first deflection of the Q-wave to the nadir of the S-wave (defined as the point of minimum voltage in the terminal portion of the QRS complex; method I, QRS1), and to the onset of the J wave (method II, QRS2) [21]. The QT interval was defined as the interval from the beginning of the QRS complex to the end of the T-wave (defined as the point where the T-wave merges with the isoelectric line). This QT value is corrected (QTc) by the Mitchell formula QT/(sqrt(RRx10)) [22]. The R-R interval was obtained as the average R-R interval over the sampling period. ECG recordings of good quality (low baseline drift, low noise, minimum number of beats to average) were used for analysis for each genotype/time point.

### 2.3. Optical Mapping

High-resolution optical mapping experiments were performed on hearts isolated from 30- to 50-week-old *Cntn2^Cre-EGFP^^/+^*; *Hand1^fx/fx^*, *Hand1^LV^-Cre*; *Hand2^fx/fx^* mice and control littermates (*Hand1^fx/fx^* and *Hand2^fx/fx^ mice)* as described previously [13,23]. Hearts were isolated and retrogradely perfused in Langendorff mode with temperature-controlled (37 °C) Krebs–Henseleit solution (pH 7.4 when gassed with a mixture of 95% O_2_ and 5% CO_2_) at an aortic pressure of 70 cm H_2_O. A volume-conducted ECG was monitored continuously throughout the experiment. After 10 min of stabilization, the hearts were stained with the voltage-sensitive dye Di-4-ANEPPS (2 μL of a 2-mmol/L stock solution). The heart was then washed with dye-free solution for 5 min followed by the addition of (±)-blebbistatin to uncouple contraction from excitation (10 µmol/L; Tocris Bioscience, Minneapolis, MN). The stained hearts were illuminated with a laser at a 532 nm wavelength and the fluorescence was collected by a MiCAMUltima-L CMOS camera (SciMedia, Costa Mesa, CA, USA) through a 715-nm long-pass filter. The fluorescence was recorded at a 1 ms/frame rate in a 100 × 100-pixel grid with a spatial resolution of 0.35 × 0.35 mm^2^ per pixel. Optical signals were processed with both spatial (3 × 3 pixels Gaussian filter) and temporal (3 frames moving average) filtering. Hearts were paced at the right atrium at a cycle length of 120 ms. Two 1 s recordings were captured sequentially while the right atrium was paced. Finally, two 1 s recordings were acquired while the hearts were paced from the ventricular apex at a cycle lengths of 120 ms [13,23].

### 2.4. Statistical Analysis

Differences between data sets were first analyzed for normal distribution by applying the Shapiro–Wilks’ test for normality. Student’s *t*-test calculator for independent means was used for all normal data sets. Data sets that failed the test for normality were analyzed using the Mann–Whitney U test (* *p* < 0.05, # *p* < 0.01). The one-way repeated measures ANOVA test was used to examine trends within groups. Groups that showed significant differences were further analyzed with pairwise *t*-test comparisons. SigmaPlot version 13 (Inpixon, Palo Alto, CA, USA) was used to conduct statistical analysis.

## 3. Results

### 3.1. Neonatal Deletion of Hand1 Does Not Observably alter CCS Function

To investigate the role of HAND1 in the post-natal CCS, we employed the *Cntn2^Cre-EGFP^* knock-in allele crossed to *Hand1^fx/fx^* mice to generate *Cntn2^Cre-EGFP^*; *Hand1^fx/fx^* mice (*H1CKO*s). Male and female littermates wherein the *Cre* allele was absent (*Hand1^fx/fx^*, *H1control*) were used as controls. We measured heart rate, PR interval, QRS1, QRS2, and QTc (Table S2). Results reveal a statistically significant reduction in heart rate in female *H1CKO*s at 5-week time point (371 bpm ± 28 vs. 422 bpm ± 9 in controls, *p* = 0.002 by Student’s *t*-test), an increase in PR interval duration in female *H1CKO*s at 25 weeks in Lead I (44.93 ms ± 3.35 vs. 39.48 ms ± 2.35 in controls, *p* = 0.0096 by Student’s *t*-test), an increase in PR interval (44.30 ms ± 4.49 vs. 38.42 ms ± 3.29 in controls, *p* = 0.022 by Student’s *t*-test) and decrease in QTc duration (37.18 ms ± 6.05 vs. 47.74 ms ± 5.83 in controls, *p* = 0.0082 by Student’s *t*-test) in female *H1CKO*s at 25 weeks in Lead II (Figure S1, Table S2). However, none of these changes are consistently observed across time points or tested parameters. Therefore, we conclude that in contrast to the observations reported from *Hand1* congenital deletion mice [15], there are no significant electrocardiographic correlations of CCS dysfunction in the parameters tested in *H1CKO*s to suggest that HAND1 is playing a role in maintaining cardiac conduction beyond the development of the CCS (Figure S1).

**Figure 1:**
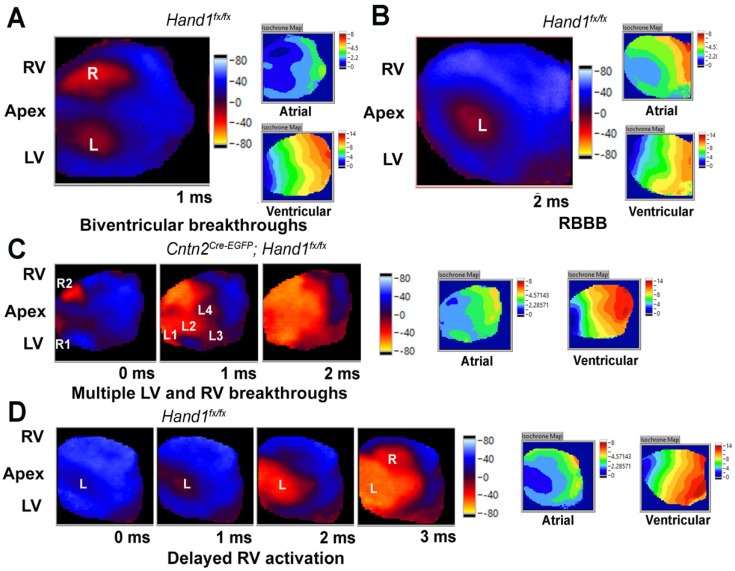
**HAND1 function is not required for post-natal CCS homeostasis.** Snapshots of representative epicardial voltage maps obtained during right atrial pacing (cycle length, 120 ms) in isolated-perfused *H1CKO* or *H1control* hearts. Isochrone activation maps during atrial and ventricular pacing (cycle lengths, 120 ms) are also shown for each heart. Number below each snapshot image is time in ms with 0 being the time of first epicardial breakthrough. Color codes for snapshots are in arbitrary units. Please note that membrane depolarization causes a decrease in the dye’s fluorescence intensity. Color codes for the activation maps are in ms. R, right; L, left. (**A**) Simultaneous and single LV (L) and RV (R) breakthroughs as expected in mature wildtype hearts. (**B**) Right BBB, left side epicardial breakthrough (L). (**C**) Multiple RV (R1, R2) and LV breakthroughs (L1–L4). (**D**) Delayed RV activation, RV epicardial breakthrough at 3 ms.

*Hand1* is robustly expressed in the myocytes of the embryonic LV when the terminal VCS is patterned [10]. Additionally, it is also established that the lack of an electrocardiographic phenotype does not rule out the possibility of a ventricular activation defect [21]. Indeed, we previously observed VCS activation defects in *Hand1^36;75/36;75^* SNP mutants that exhibited no statistically significant variations in ECG parameters [15]. Therefore, to determine ventricular activation patterns in *H1CKO*s, we performed epicardial optical voltage mapping of isolated perfused, atrially paced hearts from five male *H1CKO*s, three male *H1controls*, three female *H1CKO*s, and two female *H1controls* between 30 and 50 weeks of age (Figure 1). Four male and two female *H1CKO* hearts as well as one male and both female controls showed epicardial breakthroughs occurring synchronously over each ventricular apex, which is expected for mature mouse hearts (Figure 1A). One male and one female *H1CKO* heart exhibited a single LV epicardial breakthrough indicative of right BBB (labeled L in Figure 1B). One female *H1CKO* heart displayed multiple sites of activation throughout the ventricles (Figure 1C, R1-R2, L1-L4), resulting in wavefront collisions within the ventricular myocardium. Two male *H1controls* also presented with right BBB or delayed (3 ms) RV breakthrough (Figure 1D). During pacing at the apex of the LV (cycle length, 120 ms), activation occurred within 10–16 ms in all 13 hearts. This range of ventricular activation times is in excellent agreement with previous reports for isolated-perfused wildtype mouse hearts [24]. Since activation maps during ventricular pacing did not reveal areas of slowed or blocked conduction, we did not perform detailed analyses of local conduction velocities. Additionally, since no robust changes in the QT_c_ interval across time were found, we did not evaluate ventricular action potential duration. Taken together, these findings do not reveal consistent differences in ventricular myocardial conduction between *H1CKO* mutants and *H1controls*, further suggesting that *Hand1* function is not required for post-natal VCS homeostasis.

### 3.2. Neonatal Deletion of Hand2 Results in Sex-Dependent Effects on CCS Function

**Figure 2:**
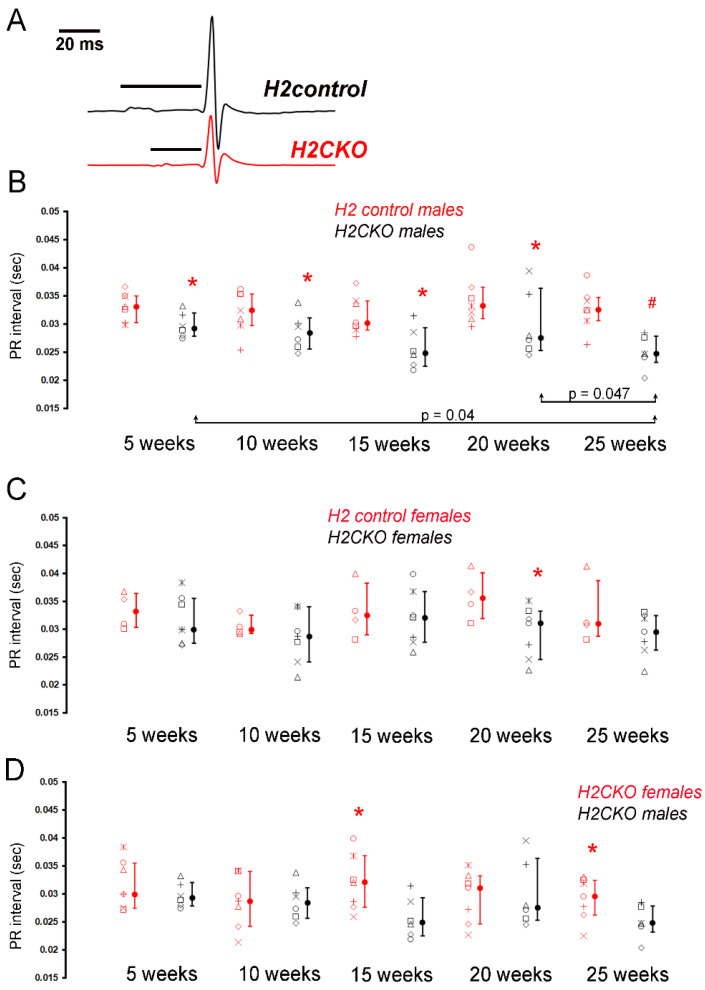
**Loss of *Hand2* in post-natal CCS results in sex-dependent effects on AV conduction.** (**A**) Exemplary surface ECGs recorded from an anesthetized *H2CKO* male (red line) and male *H2control* littermate (black line) at 25 weeks of age. Horizontal black lines above traces denote lengths of PQ intervals. (**B**) PR intervals (in seconds) in Lead I ECG analysis from male *Cntn2^Cre-EGFP/+^; Hand2^fx/fx^* (*H2CKO*s, open black symbols, *n* = 7) and *Hand2^fx/fx^* (*H2control*, open red symbols, *n* = 6) mice at 5-week, 10-week, 15-week, 20-week, and 25-week time points. Identical symbols on the left of each line in graph represent the values of the same individual mouse across time points. Filled circles, medians; error bars, 25th and 75th percentiles. * *p* < 0.05, # *p* < 0.01 versus male *H2control*. (**C**) PR intervals (in seconds) in Lead I ECG analysis from female *Cntn2^Cre-EGFP/+^; Hand2^fx/fx^* (*H2CKO*s, open black symbols, *n* = 7) and *Hand2^fx/fx^* (*H2control*, open red symbols, *n* = 4) mice at 5-week, 10-week, 15-week, 20-week, and 25-week time points. Identical symbols on the left of each line in graph represent the values of the same individual mouse across time points. Filled circles, medians; error bars, 25th and 75th percentiles. * *p* < 0.05 versus female *H2control*. (**D**) PR intervals (in seconds) in Lead I from male (open black symbols) and female (open red symbols) *H2CKO* mice at 5-week, 10-week, 15-week, 20-week, and 25-week time points. Identical symbols on the left of each line in graph represent the values of the same individual mouse across time points. Filled circles, medians; error bars, 25th and 75th percentiles. * *p* < 0.05 versus female *H2CKO*.

To investigate the role of HAND2 in the post-natal CCS, we bred the *Cntn2^Cre-EGFP/+^* knockin *Cre* line to *Hand2^fx/fx^* mice to generate *Cntn2^Cre-EGFP/+^*; *Hand2^fx/fx^* mice (*H2CKO*s) and compared these to littermate *Hand2^fx/fx^* (*H2control*) mice. We observe statistically significant shortening in Lead I PR interval in *H2CKO*s males at 5-, 10-, 15-, 20-, 25-week time points (Figure 2A,B, Table S3). In Lead II, we observe statistically significant shortening in PR interval at 5–20-week time points in *H2CKOs* males compared to *H2control* males (Figure S2). No changes were observed in QRS1, QRS2, and QTc in male *H2CKO*s in Lead I and Lead II (Figure S2, Table S3). Heart rates in *H2CKO*s are decreased at 10 weeks (517 bpm ± 28, *p* = 0.021 by Student’s *t*-test) and 20 weeks (521 bpm ± 23, *p* = 0.045 by Student’s *t*-test) compared to *H2controls* (550 bpm ± 27 at 10 weeks and 550 bpm ± 35 at 20 weeks); however, at the three other remaining time points examined, heart rates between *H2CKO*s and *H2control*s exhibit no statistically significant differences, suggesting that the consistent shortening of PR-interval phenotype is independent of changes in heart rate.

Comparison of female *H2CKO*s and *H2controls* also revealed a statistically significant shortening of Lead I QRS1 duration at 5 weeks (7.08 ms ± 0.70 vs. 8.30 ms ± 0.34 in control, *p* = 0.0081 by Student’s *t*-test) and shortening in PR interval at 20 weeks (29.38 ms ± 4.64 vs. 35.87 ± 4.31 in control, *p* = 0.05 by Student’s *t*-test, Figure 2C, Table S3). In Lead II, *H2CKO* females at 10 weeks exhibited longer QRS1 duration compared to *H2controls* (9.26 ms ± 1.58 vs. 7.27 ms ± 0.23 in controls, *p* = 0.015 by Student’s *t*-test). In *H2CKO*s females, at 20 weeks, PR interval is significantly shorter in Lead II (30.01 ms ± 5.81 vs. 37.07 ms ± 5.19 in controls, *p* = 0.039 by Student’s *t*-test). QRS1 and QRS2 duration in the same group is also increased compared to *H2controls* (QRS1: 8.58 ms ± 0.71 vs. 7.23 ms ± 0.18 in controls, *p* = 0.0022 by Student’s *t*-test, QRS2: 11.10 ms ± 1.01 vs. 9.51 ms ± 0.39 in controls, *p* = 0.0069 by Student’s *t*-test, Table S3). No significant changes in heart rate are observed between female *H2CKO*s and *H2control*s (Figure S2).

It is noteworthy that, similar to our observation in males, the PR interval in female *H2CKOs* is shorter at all time points analyzed in both Lead I and Lead II (Table S3). To examine the role that sex might play in the reduction of PR interval, we compared male and female Lead I PR values from *H2CKOs* (Figure 2D, Table S3). At 5-week, 10-week and 20-week time points, there is no significant difference in PR interval; however, at 15- and 25-week time points, PR interval in *H2CKO* males are shorter (Figure 2D). We compared the morphology of the P-wave in these recordings and do not observe a variation over time points (data not shown). To determine trends within groups and individual mice, we performed a one-way repeated ANOVA on the data (Table S6). Results show that there does not appear to be a simple progressive decrease in the PR interval within individual *H2CKO*s. However, pairwise multiple comparisons using the Student–Newman–Keul method indicate statistically significant changes between the 5-week and 25-week time points, and between the 20-week and 25-week time points (Figure 2B, Table S6), suggesting a worsening of the PR interval shortening phenotype over time. This would suggest a homeostatic role for *Hand2* in the regulation of atrioventricular conduction. Thus, loss of *Hand2* in post-natal conduction system leads to shortened PR interval in males, and females exhibit similar phenotype albeit less severe, suggesting defective atrioventricular conduction.

### 3.3. Neonatal Deletion of Hand1 and Hand2 Results in an Intermediate CCS Phenotype

**Figure 3:**
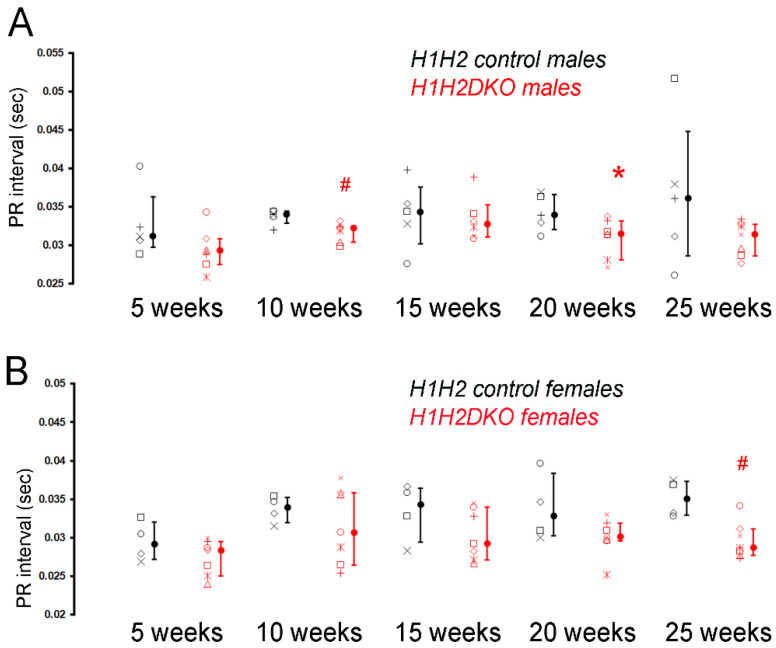
**Loss of *Hand1* and *Hand2* in post-natal CCS results in intermediate phenotype.** (**A**) PR intervals (in seconds) in Lead I ECG analysis from male *Cntn2^Cre-EGFP/+^; Hand1^fx/fx^Hand2^fx/fx^* (*H1H2DKO*s, open red symbols, *n* = 7) and *Hand1^fx/xf^Hand2^fx/fx^* (*H1H2control*, open black symbols, *n* = 5) male mice at 5-week, 10-week, 15-week, 20-week, and 25-week time points. Identical symbols on the left of each line in graph represent the values of the same individual mouse across time points. Filled circles, medians; error bars, 25th and 75th percentiles. * *p* < 0.05, # *p* < 0.01 versus male *H1H2control*. (**B**) PR intervals (in seconds) in Lead I ECG analysis from female *Cntn2^Cre-EGFP/+^; Hand1^fx/fx^Hand2^fx/fx^* (*H1H2DKO*s, open red symbols, *n* = 7) and *Hand1^fx/xf^Hand2^fx/fx^* (*H1H2control*, open black symbols, *n* = 4) female mice at 5-week, 10-week, 15-week, 20-week, and 25-week time points. Identical symbols on the left of each line in graph represent the values of the same individual mouse across time points. Filled circles, medians; error bars, 25th and 75th percentiles. # *p* < 0.01 versus female *H1H2control*.

Considering the variable phenotypes observed when *Hand1* or *Hand2* are deleted in the post-natal conduction system, we proceeded to test whether loss of both HAND factors resulted in changes to CCS. We intercrossed *Cntn2^Cre-EGFP/+^* and *Hand1^fx/fx^Hand2^fx/fx^* mice to generate *Cntn2^Cre-EGFP/+^*; *Hand1^fx/fx^Hand2^fx/fx^* mice (*H1H2DKO*s) and littermate *Hand1^fx/fx^Hand2^fx/fx^* (*H1H2controls*). We observe a statistically significant decrease in male PR interval duration in Lead I at 10-week and 20-week time points (Figure 3A, Table S4). PR interval in *H1H2DKO* is shortened in Lead I at other time points compared to *H1H2controls*, although it does not reach significance (Table S4, Figure S3). We also observe that the shortening of PR interval in *H1H2DKO* is not as severe as observed in *H2CKOs* (Tables S3 and S4). In Lead I, *H1H2DKO*s QRS1 is significantly lengthened compared to controls in 25-week males. In Lead II, *H1H2DKO*s show increased QRS1 duration at 15 weeks compared to *H1H2controls*. However, these QRS defects observed in *H1H2DKO*s are not consistently seen across all time points, and thus, might reflect an experimental artifact.

In female *H1H2DKO*s, the PR interval in Lead I and Lead II trends shorter at all time points and is significantly shortened at the 25-week time point (Figure 3B, Table S4). Lead II measurements of females also shows significantly longer QRS2 and QTc interval at 15-week compared to *H1H2controls*, although this trend is not maintained across all time points (Table S4). Repeated one-way ANOVA testing did not demonstrate any progressive changes in PR interval (data not shown). Thus, loss of both *Hand* genes within the post-natal CCS results in some significant conduction parameters changes, but there are no consistent trends in these data.

### 3.4. Embryonic Deletion of Hand2 within the LV Results in Increase in PR Interval and Arrhythmias

Although HAND2 is predominantly expressed in the endocardium [25] of the developing heart, myocytes of the embryonic RV and LV also express *Hand2* and LV deletion of *Hand2* shows dramatic phenotypic influence when *Hand1* is similarly deleted [17,25]. To test whether loss of *Hand2* in the developing embryonic LV myocardium leads to VCS defects, we intercrossed *Hand1^LV^-Cre* with *Hand2^fx/fx^* to generate *Hand1^LV^-Cre*; *Hand2^fx/fx^* (*H2LVCreCKO*s) and littermate *H2^fx/fx^* (*H2control*s). In contrast to neonatal CCS *Hand2* deletion, *H2LVCreCKO*s males exhibit statistically significantly longer PR intervals at 10 weeks and 15 weeks compared to *H2controls* in Lead I (Table S5). At week 10, *H2LVCreCKO* males in Lead I also reveal a significantly longer QRS2 duration (Table S5). At 25 weeks, *H2LVCreCKO*s males exhibit a statistically significant reduction in heart rate. Female *H2LVCreCKO*s have statistically significant longer QTc interval at week 10 in Lead I and at week 5 in Lead II; however, this phenotype was not consistent at other time points (Figure S4, Table S5).

**Figure 4:**
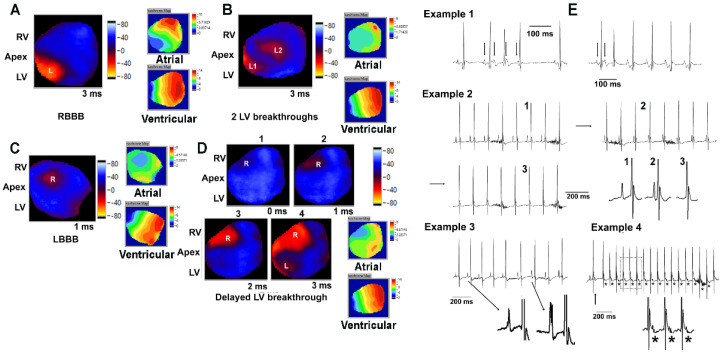
**Loss of *Hand2* in embryonic CCS results in ventricular conduction defects and atrial arrhythmias.** Snapshots of representative epicardial voltage maps obtained during right atrial pacing (cycle length, 120 ms) in isolated-perfused *H2LVCreCKO* or *H2control* hearts. Isochrone activation maps during atrial and ventricular pacing (cycle lengths, 120 ms) are also shown for each heart. Number below each snapshot image is time in ms with 0 being the time of first epicardial breakthrough. Color codes for snapshots are in arbitrary units. Please note that membrane depolarization causes a decrease in the dye’s fluorescence intensity. Color codes for the activation maps are in ms. R, right; L, left. (**A**) Right BBB, only left side epicardial breakthrough (L). (**B**) Two LV breakthrough sites (L1, L2). (**C**) LBBB, only right side epicardial breakthrough (R). (**D**) Several millisecond delay between RV and LV activation. (**E**) Examples of spontaneous arrhythmias in *H2LVCreCKO* mice. Example 1, left panel: short run of atrial tachycardia with Wenckebach-type AV conduction block (left; first arrow denotes sinus beat whereas the following three arrows indicate atrial premature beats exhibiting a P wave morphology that is distinctly different from that during sinus rhythm. The third premature beat is not conducted to the ventricles). Right: A sinus beat (first arrow) is followed by an atrial premature beat (second arrow) initiating a sustained atrial tachycardia. Note difference in P wave morphology before from that during tachycardia. Example 2: progressive shortening of PR interval until the P wave is eventually embedded in the QRS complex and undetectable on the ECG. The sinus node functions as a pacemaker for the atria but slowed conduction across the AV node allows a junctional pacemaker located between the AV node and the origin of the His bundle branches to emerge and drive the associated QRS complex, i.e., junctional rhythm. Left lower panel shows zoom-in views of the correspondingly numbered cycles in the low-resolution traces. Example 3: sudden change in P wave morphology, indicative of a competing ectopic atrial pacemaker. Example 4: sustained supraventricular tachycardia with 1:1 AV conduction. Asterisks (*) denote P waves. The tachycardia is initiated by an atrial premature contraction (arrow). Lower trace shows zoom-in views of 3 consecutive cycles framed by the dotted box.

Next, we functionally evaluated the CCS of *H2LVCreCKO*s by optically mapping 41 *H2LVCreCKO* hearts (14 male hearts) and 16 *H2control* hearts (seven male hearts; Figure 4). Maps were obtained during atrial or ventricular pacing at a cycle length of 120 ms. In each heart, we measured the interval between right atrial depolarization and first ventricular breakthrough during atrial pacing, the number of right and left ventricular breakthrough sites, the prevalence of BBB, the delay between the right and left ventricular breakthroughs (in hearts without bundle branch block), and the ventricular activation time during pacing at the LV apex. Exemplary voltage maps are illustrated in Figure 4A–D, and the results are summarized in Table S6. While the prevalence of BBB was not significantly different between female (10 out of 36 hearts) and male hearts (4 out of 21 hearts; *p* > 0.05 by *Fisher Exact* test), female hearts exhibited a higher prevalence of right BBB (*p* = 0.04 by Fisher Exact test). Numbers of breakthrough sites were similarly distributed between sexes and genotypes, with the majority of hearts exhibiting a single breakthrough on either or each ventricular apex. In male *H2LVCreCKO* hearts without BBB, there was a significant delay between occurrences of epicardial breakthroughs compared to *H2control* hearts (Table S6). During ventricular pacing at the LV apex (cycle length, 120 ms), activation occurred within 10 to 16 ms in all 57 hearts with no local conduction slowing or block being apparent on epicardial voltage maps. The average time interval from onset of right atrial depolarization to the first ventricular epicardial breakthrough in hearts without BBB was significantly longer in *H2LVCreCKO* hearts of either sex compared to their respective control hearts (see Table S6). Since isochrone activation maps and surface electrocardiograms did not reveal ventricular conduction or repolarization abnormalities, respectively, we did not perform detailed analyses of local conduction velocities or action potential duration. Four *H2LVCreCKO* mice of either sex, but none of *H2controls*, displayed arrhythmias, manifesting as supraventricular tachycardia with Wenckebach-type or 1:1 AV conduction (examples 1 and 4, respectively, in Figure 4E), junctional rhythm (example 2 in Figure 4E), or varying P wave morphologies (example 3 in Figure 4E).

Overall, our findings indicate that embryonic loss of cardiac *Hand2* expression predisposes to arrhythmias and causes abnormal conduction across the CCS.

## 4. Discussion

Anomalies in cardiac rhythm when encountered and untreated are known to lead to sudden death [5]. The CCS specifies and differentiates from existing cardiomyocytes via the interactions of several transcription factors within the embryonic heart [7]. We recently discovered that HAND1 plays an important congenital role in the formation and function of the VCS [15] and the goal of this study was to interrogate HAND1 function after the CCS is formed and to determine if HAND2 plays a similar or unique role in CCS function. We can conclude from *Hand1* neonatal deletion *Cntn2^Cre-EGFP^*; *Hand1^fx/fx^* (*H1CKO*s) that HAND1 does not appear to be influencing CCS homeostasis in any significant way. Although we do observe a longer PR interval duration on ECG at 25-week in *H1CKO* females and BBB in some males and females when optically mapped, these phenotypes are not consistent over the evaluation period making these mice indistinguishable from controls (Figure 1 and Table S1). It is possible that *Hand1^fx/fx^* mice could also present with CCS phenotypes that could be confounding our analysis. However, when compared to *Nkx2.5^Cre/+^;Hand1^fx/fx^* [13], *Hand1^ΔLV/^^ΔLV^* or *Hand1^36;75/36;75^* [15], significant phenotypes are statistically evident when utilizing *Hand1^fx/fx^* mice for control comparisons. Based on these cumulative data, we conclude that under normal non-pathological conditions, the neonatal loss of HAND1 does not pathologically alter murine CCS function.

Interestingly, neonatal deletion of *Hand2*, *Cntn2^Cre-EGFP^*; *Hand2^fx/fx^* (*H2CKO*s) results in a shorter PR interval compared to controls (Figure 2). Earlier studies revealed that HAND2 influenced the formation of pacemaker like cells from fibroblast differentiation analysis [14]. Shortening of PR interval can occur due to a change in location of the atrial pacemaker such that the time from impulse generation to AVN excitation is shortened as a result of the smaller distance between the pacemaker and AVN. However, this is an unlikely mechanism here, as the neonatal deletion of *Hand2* occurs well after CCS morphogenesis is complete. Another possible cause for shortening of PR interval is an acceleration of conduction across the bundle of His. Pre-excitation syndromes such as Wolf–Parkinson–White can also cause a shortening of PR-interval [26]. However, we can rule out the existence of accessory atrioventricular conduction pathways as underlying mechanism because neither atrial nor ventricular pacing at incremental rates in our optical mapping experiments revealed evidence for propagation across AVN-bypassing tracts. It is interesting that shortening of the PR interval is also observed in *Hand1^ΔLV/^^ΔLV^* mice [15] as well as in *Gata4^+/-^* mice [27]. Evidence suggests GATA4 is an upstream transcriptional regulator of HAND1 (binding to two evolutionarily conserved GATA *cis*-elements within the *Hand1* LV-enhancer) [15], as well as regulating *Hand2* RV expression [28]. Embryonic LV deletion of both *Hand1* and *Hand2* exhibit single ventricle like phenotypes with compromised cardiac function, but the extensive morphological phenotypes would confound any clear conclusions from measure of conduction parameters [17].

Given the significant variations in ECG phenotypes observed in *H1CKOs* and *H2CKO*s, we generated conditional knockouts of both HAND factors with the *Cntn2^Cre-EGFP**/+**^*; *Hand1^fx/fx^ Hand2^fx/fx^* (*H1H2CKO*s) allele to test if loss of both factors in the post-natal CCS had an additive or reductive effect on CCS function (Figure 3, Table S3). We observe significant reduction in PR interval duration in males and females at the 10-week and 20-week time point. However, this phenotype is not as severe or as consistent as observed in *H2CKO*s, suggesting some rescue of the *H2CKO* phenotype from the loss of the *Hand1* allele.

Surprisingly, embryonic *Hand2* deletion using the *Hand1^LV^-Cre* revealed a longer PR interval on ECG (Table S5), as well as longer delays between onset of right atrial depolarization and first epicardial breakthrough in optical voltage maps (Table S7). At first glance this seems odd; however, it is possible that the loss of HAND2 impacts CCS morphogenesis such that distance between the atrial pacemaker and AVN is increased. We currently do not have evidence of this possibility, but it would explain the variation from the neonatal results obtained from *Cntn2^Cre-EGFP**/+**^* mediated deletion. Notably; however, some adult *H2LVCreCKO* mice have varying P-wave morphology and atrial tachycardia (Figure 4), suggesting that embryonic loss of cardiac *Hand2* expression alters atrial electrical properties, increasing their arrhythmia susceptibility. Atrial arrhythmias result from increased automaticity, triggered activity arising from afterdepolarizations, re-entry electrical activity, or a combination of these. Since afterdepolarizations were never observed in atrial voltage maps, they are unlikely to underlie arrhythmia in *H2LVCreCKO* mice. Whether the other mechanisms contributed to atrial arrhythmogenesis remains to be determined.

HAND1 and HAND2 are robustly expressed in the sympathetic nervous system (SNS), derived from migrating cardiac neural crest cells, and HAND2 is required for SNS development and function [29,30,31,32]. Functional changes in the AV conduction system can be caused by the action of the autonomic nervous system [33]. It is possible that loss of HAND factors within the post-natal cardiac autonomous nervous system could cause the observed time-point specific variations in the CCS system.

The bHLH transcription factor MYOR is expressed within the AVN during development and represses GATA4-dependent activation of the Cx30.2 enhancer [34]. HAND2 and GATA are established to physically interact to co-regulate transcription [35]. Given that Cx30.2 knockout mice also exhibit a shortened PR interval, by accelerating suprahissian impulse propagation [36], an interesting mechanism of action could be that HAND2 and GATA4 act at the Cx30.2 enhancer where loss of GATA4 or HAND2 results in a reduction in PR interval.

Another interesting observation is that we observe a sex-dependent effect on loss of HAND factors on the CCS, with a more severe phenotype being observed in male mutants compared to females (Figure 2D, Tables S3 and S7, Figure S5). Although interesting, it is currently unclear what role sex plays in HAND-factor-mediated developments/maintenance of the CCS.

Overall, these experiments suggest that although HAND1 plays a critical role in CCS formation and morphogenesis, its deletion from the CCS after it is formed does not appear to significantly alter function. In contrast, HAND2 does appear to play a homeostatic role in maintaining the CCS, as HAND2 loss-of-function within the post-natal CCS results in abnormalities in AV conduction. Loss of both HAND alleles in the post-natal CCS results in an intermediate phenotype. This is an intriguing result as we would predict that loss of both factors would lead to a worse phenotype, suggesting that loss of HAND1 rescues the shortened PR-interval HAND2 phenotype. Further, our results support a role of HAND2 expression in the embryonic heart for CCS formation and development as *H2LVCreCKO* mice exhibit abnormalities in conduction across the cardiac conduction system.

## Data Availability

Not applicable.

## Supplementary Materials

The following supporting information can be downloaded at: https://www.mdpi.com/article/10.3390/jcdd9070214/s1, Table S1: Alleles used for experiments; Figure S1 and Table S2: Column graphs of ECG Analysis of *Cntn2^Cre-EGFP/+^*; *Hand1^fx/fx^* (*H1CKO*s) and Table of ECG Analysis of *Cntn2^Cre-EGFP/+^*; *Hand1^fx/fx^* (*H1CKO*s); Figure S2 and Table S3: Column graphs of ECG Analysis of *Cntn2^Cre-EGFP/+^*; *Hand2^fx/fx^* (*H2CKO*s) and Table of ECG Analysis of *Cntn2^Cre-EGFP/+^*; *Hand2^fx/fx^* (*H2CKO*s); Figure S3 and Table S4: Column graphs of *Cntn2^Cre-EGFP/+^*; *Hand1^fx/fx^Hand2^fx/fx^* (*H1H2CKO*s) and Table of ECG Analysis of *Cntn2^Cre-EGFP/+^*; *Hand1^fx/fx^Hand2^fx/fx^* (*H1H2CKO*s); Figure S4 and Table S5: Column graphs of ECG Analysis of *Hand1^LV^Cre*; *Hand2^fx/fx^* (*H2LVCreCKO*) and Table of ECG Analysis of *Hand1^LV^Cre*; *Hand2^fx/fx^* (*H2LVCreCKO*); Table S6: Results of repeated one-way ANOVA on *Cntn2^Cre-EGFP/+^*; *Hand2^fx/fx^* males; Table S7: Results of epicardial optical voltage mapping of adult *H2LVCreCKO* and *H2control* hearts; Table S8: ARRIVE guidelines; Figure S5: Comparison of male vs female controls.
