# Supplementary material for: Neonatal Deletion of Hand1 and Hand2 within Murine Cardiac Conduction System Reveals a Novel Role for HAND2 in Rhythm Homeostasis
Source: J Cardiovasc Dev Dis. 2022 Jul 4;9(7):214. doi: 10.3390/jcdd9070214 (PMC9324487; doi:10.3390/jcdd9070214)
Supplement: Supplementary file 1 [file jcdd-09-00214-s001.zip › Table S1.pdf]

Table S1: Table of Alleles used for experiments

Allele	Abbreviated Allele	Reference
Cntn2$^{3'UTR\text{-}IRES\text{-}Cre\text{-}EGFP}$	*Cntn2*$^{Cre\text{-}EGFP/+}$	Bhattacharyya, S., M. Bhakta, and N.V. Munshi, *Phenotypically silent Cre recombination within the postnatal ventricular conduction system.* PLoS One, 2017. **12**(3): p. e0174517
Hand1^{LV}*-Cre*	*Hand1*^{LV}*-Cre*	Vincentz, J.W., et al., *Hand factor ablation causes defective left ventricular chamber development and compromised adult cardiac function.* PLoS Genet, 2017. **13**(7): p. e1006922
Hand1^{tm2Eno}	*Hand1*$^{fx/fx}$ (*H1control*)	McFadden, D.G., et al., *The Hand1 and Hand2 transcription factors regulate expansion of the embryonic cardiac ventricles in a gene dosage-dependent manner.* Development, 2005. **132**(1): p. 189-201
Hand2^{tm1Cse}	*Hand2*$^{fx/fx}$ (*H2control*)	Morikawa, Y. and P. Cserjesi, *Cardiac neural crest expression of Hand2 regulates outflow and second heart field development.* Circ Res, 2008. **103**(12): p. 1422-9
Hand1$^{fx/fx}$ *Hand2*$^{fx/fx}$	*H1H2control*	
Cntn2$^{Cre\text{-}EGFP/+}$; *Hand1*$^{fx/fx}$	*H1CKO*	
Cntn2$^{Cre\text{-}EGFP/+}$; *Hand2*$^{fx/fx}$	*H2CKO*	
Cntn2$^{Cre\text{-}EGFP/+}$; *Hand1*$^{fx/fx}$; *Hand2*$^{fx/fx}$	*H1H2CKO*	
Hand1^{LV}*-Cre; Hand2*$^{fx/fx}$	*H2LVCreCKO*	
