# Supplementary material for: Neonatal Deletion of Hand1 and Hand2 within Murine Cardiac Conduction System Reveals a Novel Role for HAND2 in Rhythm Homeostasis
Source: J Cardiovasc Dev Dis. 2022 Jul 4;9(7):214. doi: 10.3390/jcdd9070214 (PMC9324487; doi:10.3390/jcdd9070214)
Supplement: Supplementary file 1 [file jcdd-09-00214-s001.zip › Table S2.pdf]

Table S2. ECG Analysis of *Cntn2*^{Cre} *H1*$^{fx/fx}$

Males - LEAD I

Age (weeks)	n	Genotype	Heart rate (bpm)	PR interval (ms)	QRS1 (ms)	QRS2 (ms)	QTc (ms)
5	8	*Cntn2*^{Cre} *H1*$^{fx/fx}$	377 ± 39	42.34 ± 3.77	8.92 ± 0.13	11.56 ± 0.10	46.88 ± 11.15
	10	*H1*$^{fx/fx}$	417 ± 57	40.32 ± 7.38	8.43 ± 0.84	11.11 ± 0.95	46.01 ± 12.48
10	8	*Cntn2*^{Cre} *H1*$^{fx/fx}$	412 ± 50	41.76 ± 5.51	9.46 ± 1.46	11.46 ± 1.35	39.36 ± 6.97
	10	*H1*$^{fx/fx}$	436 ± 51	40.63 ± 5.29	9.29 ± 0.96	11.58 ± 1.02	44.16 ± 5.76
15	9	*Cntn2*^{Cre} *H1*$^{fx/fx}$	454 ± 103	41.63 ± 2.79	9.43 ± 1.13	11.24 ± 0.81	39.96 ± 8.84
	10	*H1*$^{fx/fx}$	468 ± 64	40.01 ± 4.63	9.35 ± 0.79	11.27 ± 1.10	45.37 ± 5.46
20	9	*Cntn2*^{Cre} *H1*$^{fx/fx}$	440 ± 56	48.11 ± 4.79	9.15 ± 0.79	11.09 ± 0.71	49.09 ± 25.33
	10	*H1*$^{fx/fx}$	474 ± 46	46.41 ± 5.16	9.16 ± 0.84	10.83 ± 0.99	43.10 ± 7.65
25	7	*Cntn2*^{Cre} *H1*$^{fx/fx}$	459 ± 69	45.22 ± 6.04	8.92 ± 0.80	11.23 ± 0.80	45.98 ± 3.51
	10	*H1*$^{fx/fx}$	465 ± 38	43.12 ± 4.97	10.06 ± 2.64	12.16 ± 2.54	47.24 ± 6.93

Males - LEAD II

Age (weeks)	n	Genotype	PR interval (ms)	QRS1 (ms)	QRS2 (ms)	QTc (ms)
5	8	*Cntn2*^{Cre} *H1*$^{fx/fx}$	42.12 ± 6.08	9.82 ± 0.11*	12.34 ± 0.91	48.66 ± 11.39
	10	*H1*$^{fx/fx}$	40.23 ± 4.48	9.13 ± 0.48	11.79 ± 0.34	44.78 ± 15.73
10	8	*Cntn2*^{Cre} *H1*$^{fx/fx}$	41.23 ± 5.27	9.74 ± 1.26	11.45 ± 1.03	38.79 ± 5.35
	10	*H1*$^{fx/fx}$	40.23 ± 6.34	9.25 ± 0.82	11.33 ± 0.55	40.06 ± 5.04
15	9	*Cntn2*^{Cre} *H1*$^{fx/fx}$	40.15 ± 3.63	9.68 ± 0.89	12.17 ± 0.84	37.94 ± 3.88
	10	*H1*$^{fx/fx}$	40.01 ± 4.17	9.49 ± 0.12	11.58 ± 0.83	39.57 ± 3.79
20	9	*Cntn2*^{Cre} *H1*$^{fx/fx}$	48.91 ± 3.13	10.54 ± 3.23	12.52 ± 3.60	42.28 ± 7.97
	10	*H1*$^{fx/fx}$	47.08 ± 6.74	8.82 ± 0.16	10.99 ± 1.56	44.29 ± 7.68
25	7	*Cntn2*^{Cre} *H1*$^{fx/fx}$	44.56 ± 4.83	9.39 ± 0.95	12.11 ± 1.27	41.02 ± 3.84
	10	*H1*$^{fx/fx}$	41.24 ± 3.29	9.92 ± 0.86	12.21 ± 1.06	39.65 ± 0.92

Females - LEAD I

Age (weeks)	n	Genotype	Heart rate (bpm)	PR interval (ms)	QRS1 (ms)	QRS2 (ms)	QTc (ms)
5	9	*Cntn2*^{Cre} *H1*$^{fx/fx}$	371 ± 28#	39.71 ± 2.94	9.14 ± 0.79	11.55 ± 1.10	46.89 ± 8.43
	4	*H1*$^{fx/fx}$	422 ± 9	36.53 ± 3.96	8.72 ± 0.91	11.13 ± 1.24	49.95 ± 15.10
10	9	*Cntn2*^{Cre} *H1*$^{fx/fx}$	412 ± 35	41.23 ± 5.91	9.31 ± 1.14	11.26 ± 1.10	51.25 ± 10.81
	4	*H1*$^{fx/fx}$	397 ± 55	39.84 ± 4.68	9.41 ± 0.65	12.23 ± 0.52	47.78 ± 9.36
15	8	*Cntn2*^{Cre} *H1*$^{fx/fx}$	390 ± 57	41.94 ± 3.71	10.34 ± 0.29	12.76 ± 2.54	52.09 ± 19.98
	4	*H1*$^{fx/fx}$	383 ± 50	40.31 ± 2.90	8.9 ± 1.10	11.69 ± 1.73	44.13 ± 5.23
20	7	*Cntn2*^{Cre} *H1*$^{fx/fx}$	446 ± 65	46.09 ± 7.26	9.99 ± 0.78	12.58 ± 1.12	50.10 ± 5.69
	4	*H1*$^{fx/fx}$	469 ± 24	44.64 ± 2.97	9.26 ± 0.92	11.39 ± 1.30	50.41 ± 2.30
25	8	*Cntn2*^{Cre} *H1*$^{fx/fx}$	469 ± 46	44.93 ± 3.35#	9.56 ± 1.53	11.89 ± 1.74	46.86 ± 7.52
	4	*H1*$^{fx/fx}$	444 ± 65	39.48 ± 2.35	10.15 ± 1.85	13.14 ± 3.23	61.46 ± 49.75

Females - LEAD II

Age (weeks)	n	Genotype	PR interval (ms)	QRS1 (ms)	QRS2 (ms)	QTc (ms)
5	9	*Cntn2*^{Cre} *H1*$^{fx/fx}$	38.55 ± 3.71	9.07 ± 1.07	11.28 ± 1.38	39.54 ± 5.64
	4	*H1*$^{fx/fx}$	36.37 ± 4.46	8.96 ± 0.42	11.53 ± 0.77	38.44 ± 3.73
10	9	*Cntn2*^{Cre} *H1*$^{fx/fx}$	40.73 ± 5.81	9.49 ± 0.92	11.95 ± 1.28	39.92 ± 6.32
	4	*H1*$^{fx/fx}$	38.23 ± 3.46	8.96 ± 0.59	11.57 ± 0.27	34.45 ± 4.69
15	8	*Cntn2*^{Cre} *H1*$^{fx/fx}$	41.66 ± 3.88	9.95 ± 0.13	12.76 ± 1.35	42.52 ± 7.83
	4	*H1*$^{fx/fx}$	40.49 ± 2.64	9.49 ± 0.11	11.51 ± 1.19	35.73 ± 5.42
20	7	*Cntn2*^{Cre} *H1*$^{fx/fx}$	48.51 ± 7.49	8.93 ± 0.81	12.09 ± 1.32	51.70 ± 21.88
	4	*H1*$^{fx/fx}$	44.61 ± 2.44	10.89 ± 3.14	14.35 ± 5.92	45.76 ± 9.94
25	8	*Cntn2*^{Cre} *H1*$^{fx/fx}$	44.30 ± 4.49*	9.08 ± 0.99	11.37 ± 1.38	37.18 ± 6.05#
	4	*H1*$^{fx/fx}$	38.42 ± 3.29	9.54 ± 0.64	11.52 ± 0.58	47.74 ± 5.83

Normality calculated with Shapiro-Wilk normality test. For normal data sets, student's T-test used to calculate significance. For not normal data sets, Mann-Whitney U test used to calculate significance.

@ Data not normal   # p<0.01   * p<0.05 vs H1control
