# Supplementary material for: Neonatal Deletion of Hand1 and Hand2 within Murine Cardiac Conduction System Reveals a Novel Role for HAND2 in Rhythm Homeostasis
Source: J Cardiovasc Dev Dis. 2022 Jul 4;9(7):214. doi: 10.3390/jcdd9070214 (PMC9324487; doi:10.3390/jcdd9070214)
Supplement: Supplementary file 1 [file jcdd-09-00214-s001.zip › Table S3.pdf]

Table S3 A: ECG analysis of $Cntn2^{Cre}\ H2^{fx/fx}$

Males - LEAD I

Age (weeks)	n	Genotype	Heart rate (bpm)	PR interval (ms)	QRS1 (ms)	QRS2 (ms)	QTc (ms)
5	7	$Cntn2^{Cre}\ H2^{fx/fx}$	517 ± 57	29.24 ± 2.49*	6.64 ± 0.61	8.94 ± 0.38*	40.04 ± 0.51
	9	$H2^{fx/fx}$	509 ± 49	33.48 ± 2.52	6.74 ± 0.39	9.42 ± 0.64	39.48 ± 0.62
10	7	$Cntn2^{Cre}\ H2^{fx/fx}$	517 ± 28*	27.72 ± 3.76*	9.13 ± 0.10	11.05 ± 0.17	33.98 ± 6.41
	8	$H2^{fx/fx}$	550 ± 27	32.34 ± 3.62	8.41 ± 0.66	10.42 ± 0.98	35.54 ± 5.40
15	6	$Cntn2^{Cre}\ H2^{fx/fx}$	532 ± 47	25.72 ± 3.66*	9.62 ± 0.12	11.62 ± 0.12	37.49 ± 7.98
	8	$H2^{fx/fx}$	525 ± 49	31.41 ± 3.24	9.02 ± 0.81	11.92 ± 0.10	40.96 ± 4.93
20	7	$Cntn2^{Cre}\ H2^{fx/fx}$	521 ± 23*	29.44 ± 5.67*	9.29 ± 1.29	11.05 ± 2.02	47.30 ± 3.49
	7	$H2^{fx/fx}$	550 ± 35	34.36 ± 4.68	9.38 ± 0.64	11.74 ± 1.23	44.09 ± 6.02
25	6	$Cntn2^{Cre}\ H2^{fx/fx}$	542 ± 67	25.01 ± 2.86#	6.72 ± 0.51	8.48 ± 0.89	38.62 ± 4.40
	7	$H2^{fx/fx}$	586 ± 44	32.76 ± 3.79	7.09 ± 0.72	9.38 ± 0.93	37.79 ± 2.37

Males - LEAD II

Age (weeks)	n	Genotype	PR interval (ms)	QRS1 (ms)	QRS2 (ms)	QTc (ms)
5	7	$Cntn2^{Cre}\ H2^{fx/fx}$	29.20 ± 2.22*	6.62 ± 0.38	9.26 ± 0.09	36.02 ± 0.15
	9	$H2^{fx/fx}$	34.59 ± 2.94	6.97 ± 0.53	9.22 ± 0.72	37.53 ± 0.36
10	7	$Cntn2^{Cre}\ H2^{fx/fx}$	26.54 ± 3.57#	8.26 ± 1.04	11.29 ± 0.18	32.74 ± 7.39
	8	$H2^{fx/fx}$	32.72 ± 3.78	7.57 ± 0.86	10.06 ± 0.93	38.49 ± 4.92
15	6	$Cntn2^{Cre}\ H2^{fx/fx}$	26.24 ± 4.59#	8.85 ± 0.88	10.84 ± 0.17	40.03 ± 2.86
	8	$H2^{fx/fx}$	31.10 ± 1.76	8.03 ± 0.76	10.84 ± 0.97	34.84 ± 6.92
20	7	$Cntn2^{Cre}\ H2^{fx/fx}$	29.99 ± 5.43*	8.72 ± 1.56	11.14 ± 2.14	45.01 ± 4.34*
	7	$H2^{fx/fx}$	35.86 ± 4.93	8.90 ± 1.26	11.36 ± 1.44	39.44 ± 5.68
25	6	$Cntn2^{Cre}\ H2^{fx/fx}$	30.86 ± 2.83	6.84 ± 0.92	9.44 ± 0.89	37.53 ± 2.75
	7	$H2^{fx/fx}$	30.82 ± 2.67	7.25 ± 0.91	9.38 ± 0.93	39.53 ± 7.75

Females - LEAD I

Age (weeks)	n	Genotype	Heart rate (bpm)	PR interval (ms)	QRS1 (ms)	QRS2 (ms)	QTc (ms)
5	8	$Cntn2^{Cre}\ H2^{fx/fx}$	492 ± 50	31.52 ± 4.08	7.08 ± 0.70#	14.84 ± 1.65	43.98 ± 7.56
	5	$H2^{fx/fx}$	535 ± 106	33.71 ± 2.98	8.30 ± 0.34	11.78 ± 1.89	48.87 ± 12.49
10	8	$Cntn2^{Cre}\ H2^{fx/fx}$	499 ± 55	28.33 ± 4.73	9.24 ± 0.82	12.07 ± 1.22	47.29 ± 5.59
	4	$H2^{fx/fx}$	532 ± 15	30.57 ± 1.83	9.00 ± 0.56	12.47 ± 2.45	46.45 ± 1.86
15	8	$Cntn2^{Cre}\ H2^{fx/fx}$	535 ± 52	31.22 ± 5.07	9.03 ± 0.85	11.89 ± 0.12	44.58 ± 6.18
	4	$H2^{fx/fx}$	569 ± 49	33.24 ± 4.94	8.32 ± 0.46	12.13 ± 0.14	43.08 ± 3.12
20	8	$Cntn2^{Cre}\ H2^{fx/fx}$	565 ± 124	29.38 ± 4.64*	8.64 ± 0.82	10.89 ± 0.75#	48.19 ± 8.21
	4	$H2^{fx/fx}$	521 ± 44	35.87 ± 4.31	8.65 ± 1.09	9.99 ± 0.71	45.61 ± 3.59
25	7	$Cntn2^{Cre}\ H2^{fx/fx}$	529 ± 69	29.04 ± 3.84	9.16 ± 1.11	11.44 ± 1.02	46.40 ± 3.39*
	4	$H2^{fx/fx}$	564 ± 92	32.82 ± 5.79	8.65 ± 0.93	11.55 ± 1.60	46.51 ± 5.55

Females - LEAD II

Age (weeks)	n	Genotype	PR interval (ms)	QRS1 (ms)	QRS2 (ms)	QTc (ms)
5	8	$Cntn2^{Cre}\ H2^{fx/fx}$	32.75 ± 3.79	6.75 ± 0.76	9.03 ± 0.88	45.16 ± 7.63
	5	$H2^{fx/fx}$	34.87 ± 3.62	6.80 ± 0.41	9.06 ± 0.64	46.31 ± 13.11
10	8	$Cntn2^{Cre}\ H2^{fx/fx}$	30.66 ± 4.87	9.26 ± 1.58*	12.04 ± 1.54	44.01 ± 5.68
	4	$H2^{fx/fx}$	34.26 ± 3.77	7.27 ± 0.23	11.01 ± 2.79	41.65 ± 3.86
15	8	$Cntn2^{Cre}\ H2^{fx/fx}$	29.54 ± 5.90	9.02 ± 2.17	11.84 ± 0.21	43.93 ± 5.48
	4	$H2^{fx/fx}$	32.54 ± 4.57	7.67 ± 0.79	11.05 ± 2.26	42.15 ± 7.45
20	8	$Cntn2^{Cre}\ H2^{fx/fx}$	30.01 ± 5.81*	8.58 ± 0.71#	11.10 ± 1.01#	46.90 ± 5.18
	4	$H2^{fx/fx}$	37.07 ± 5.19	7.23 ± 0.18	9.51 ± 0.39	43.53 ± 2.84
25	7	$Cntn2^{Cre}\ H2^{fx/fx}$	29.32 ± 5.19	8.29 ± 0.57*	10.84 ± 0.58*	45.67 ± 3.79
	4	$H2^{fx/fx}$	33.81 ± 6.18	7.58 ± 0.61	9.65 ± 0.62	43.09 ± 2.86

Normality calculated with Shapiro-Wilk normality test. For normal data sets, student's T-test used to calculate significance. For not normal data sets, Mann-Whitney U test used to calculate significance.

@ Data not normal # $p<0.01$ * $p<0.05$ vs H2control
