# Supplementary material for: Neonatal Deletion of Hand1 and Hand2 within Murine Cardiac Conduction System Reveals a Novel Role for HAND2 in Rhythm Homeostasis
Source: J Cardiovasc Dev Dis. 2022 Jul 4;9(7):214. doi: 10.3390/jcdd9070214 (PMC9324487; doi:10.3390/jcdd9070214)
Supplement: Supplementary file 1 [file jcdd-09-00214-s001.zip › Table S4.pdf]

Table S4: ECG Analysis of $Cntn2^{Cre} H1^{fx/fx} H2^{fx/fx}$

Males - LEAD I

Age (weeks)	n	Genotype	Heart rate (bpm)	PR interval (ms)	QRS1 (ms)	QRS2 (ms)	QTc (ms)
5	7	$Cntn2^{Cre} H1^{fx/fx} H2^{fx/fx}$	447 ± 52	29.42 ± 2.65	9.49 ± 0.69	13.49 ± 2.29	48.84 ± 5.37
	5	$H1^{fx/fx} H2^{fx/fx}$	502 ± 30	32.63 ± 4.44	9.35 ± 0.69	13.44 ± 1.98	54.82 ± 9.74
10	7	$Cntn2^{Cre} H1^{fx/fx} H2^{fx/fx}$	504 ± 50	31.77 ± 1.20#	8.14 ± 0.58	11.21 ± 0.15	42.09 ± 5.85
	5	$H1^{fx/fx} H2^{fx/fx}$	513 ± 43	33.71 ± 1.01	7.56 ± 0.51	10.24 ± 1.13	39.69 ± 5.49
15	6	$Cntn2^{Cre} H1^{fx/fx} H2^{fx/fx}$	517 ± 56	33.39 ± 2.91	7.85 ± 0.55	11.36 ± 1.96	37.41 ± 7.08
	5	$H1^{fx/fx} H2^{fx/fx}$	527 ± 51	33.96 ± 4.43	7.41 ± 0.61	10.58 ± 1.21	38.09 ± 2.64
20	7	$Cntn2^{Cre} H1^{fx/fx} H2^{fx/fx}$	578 ± 41	30.94 ± 2.47*	8.07 ± 1.03	10.68 ± 1.42	38.32 ± 7.59
	5	$H1^{fx/fx} H2^{fx/fx}$	541 ± 61	34.25 ± 2.37	8.39 ± 1.52	10.92 ± 2.22	42.65 ± 13.25
25	7	$Cntn2^{Cre} H1^{fx/fx} H2^{fx/fx}$	596 ± 65	30.83 ± 2.24	8.49 ± 0.59*	11.11 ± 2.05	40.30 ± 4.66
	5	$H1^{fx/fx} H2^{fx/fx}$	563 ± 50	36.59 ± 9.60	7.67 ± 0.80	10.34 ± 1.03	39.41 ± 4.95

Males - LEAD II

Age (weeks)	n	Genotype	PR interval (ms)	QRS1 (ms)	QRS2 (ms)	QTc (ms)
5	7	$Cntn2^{Cre} H1^{fx/fx} H2^{fx/fx}$	29.37 ± 2.84	8.58 ± 0.87	12.84 ± 2.47	48.80 ± 6.42
	5	$H1^{fx/fx} H2^{fx/fx}$	31.54 ± 3.90	8.37 ± 0.94	12.73 ± 2.26	47.70 ± 7.18
10	0	$Cntn2^{Cre} H1^{fx/fx} H2^{fx/fx}$	31.96 ± 0.99	7.69 ± 0.88	10.23 ± 0.73	41.81 ± 6.04
	0	$H1^{fx/fx} H2^{fx/fx}$	31.48 ± 4.00	6.99 ± 0.63	9.88 ± 0.49	40.64 ± 5.78
15	6	$Cntn2^{Cre} H1^{fx/fx} H2^{fx/fx}$	33.23 ± 3.45	7.62 ± 0.62*	10.86 ± 1.84	45.92 ± 3.78
	5	$H1^{fx/fx} H2^{fx/fx}$	34.10 ± 1.30	6.91 ± 0.57	9.48 ± 0.71	39.56 ± 1.27
20	7	$Cntn2^{Cre} H1^{fx/fx} H2^{fx/fx}$	31.55 ± 4.96	7.43 ± 0.67	10.09 ± 1.50	41.35 ± 3.77
	5	$H1^{fx/fx} H2^{fx/fx}$	35.72 ± 6.02	7.37 ± 0.49	9.39 ± 0.46	43.91 ± 4.93
25	7	$Cntn2^{Cre} H1^{fx/fx} H2^{fx/fx}$	34.24 ± 3.19	7.22 ± 0.13	10.13 ± 0.18	43.37 ± 5.43#
	5	$H1^{fx/fx} H2^{fx/fx}$	40.23 ± 10.32	6.72 ± 0.17	8.93 ± 0.31	32.32 ± 1.63

Females - LEAD I

Age (weeks)	n	Genotype	Heart rate (bpm)	PR interval (ms)	QRS1 (ms)	QRS2 (ms)	QTc (ms)
5	7	$Cntn2^{Cre} H1^{fx/fx} H2^{fx/fx}$	452 ± 60	27.44 ± 2.30	9.81 ± 0.11	12.74 ± 2.08	47.74 ± 5.37
	4	$H1^{fx/fx} H2^{fx/fx}$	396 ± 32	29.49 ± 2.56	10.17 ± 0.89	13.21 ± 2.09	49.53 ± 2.69
10	7	$Cntn2^{Cre} H1^{fx/fx} H2^{fx/fx}$	436 ± 79	31.58 ± 4.56	7.93 ± 0.75	8.14 ± 3.11	40.10 ± 6.51
	4	$H1^{fx/fx} H2^{fx/fx}$	449 ± 33	33.70 ± 1.71	7.94 ± 0.55	10.47 ± 1.37	38.28 ± 5.69
15	7	$Cntn2^{Cre} H1^{fx/fx} H2^{fx/fx}$	487 ± 40*	30.36 ± 3.30	7.32 ± 1.06	9.84 ± 1.58	41.71 ± 2.66
	4	$H1^{fx/fx} H2^{fx/fx}$	442 ± 15	33.41 ± 3.77	9.54 ± 1.32	9.50 ± 1.70	43.91 ± 6.57
20	6	$Cntn2^{Cre} H1^{fx/fx} H2^{fx/fx}$	508 ± 56	30.06 ± 2.72	7.45 ± 0.92	9.38 ± 0.74	39.36 ± 12.56
	4	$H1^{fx/fx} H2^{fx/fx}$	523 ± 60	33.82 ± 4.35	7.85 ± 1.06	10.17 ± 1.05	39.09 ± 3.78
25	7	$Cntn2^{Cre} H1^{fx/fx} H2^{fx/fx}$	531 ± 55	29.66 ± 2.39#	7.98 ± 1.14	9.72 ± 0.76*	42.95 ± 6.33
	4	$H1^{fx/fx} H2^{fx/fx}$	527 ± 64	35.11 ± 2.41	7.85 ± 0.43	10.84 ± 0.86	40.82 ± 5.47

Females - LEAD II

Age (weeks)	n	Genotype	PR interval (ms)	QRS1 (ms)	QRS2 (ms)	QTc (ms)
5	7	$Cntn2^{Cre} H1^{fx/fx} H2^{fx/fx}$	28.30 ± 4.02	8.44 ± 1.20	11.60 ± 2.30	46.28 ± 7.58
	4	$H1^{fx/fx} H2^{fx/fx}$	31.66 ± 2.51	8.27 ± 1.02	12.46 ± 3.17	47.15 ± 6.24
10	7	$Cntn2^{Cre} H1^{fx/fx} H2^{fx/fx}$	31.39 ± 3.75	7.47 ± 0.62	9.69 ± 1.05	38.74 ± 4.19
	4	$H1^{fx/fx} H2^{fx/fx}$	34.69 ± 0.68	7.02 ± 0.11	9.94 ± 1.34	36.56 ± 8.13
15	7	$Cntn2^{Cre} H1^{fx/fx} H2^{fx/fx}$	31.09 ± 3.25	7.17 ± 0.73	9.01 ± 1.07*	39.74 ± 3.98*
	4	$H1^{fx/fx} H2^{fx/fx}$	33.83 ± 4.07	7.73 ± 0.69	7.00 ± 0.02	45.64 ± 3.55
20	6	$Cntn2^{Cre} H1^{fx/fx} H2^{fx/fx}$	31.35 ± 2.22	6.33 ± 1.16	8.55 ± 0.77	39.45 ± 3.49
	4	$H1^{fx/fx} H2^{fx/fx}$	34.41 ± 3.55	7.14 ± 0.10	9.27 ± 0.35	38.11 ± 2.73
25	7	$Cntn2^{Cre} H1^{fx/fx} H2^{fx/fx}$	30.55 ± 2.47#	7.96 ± 0.86	10.39 ± 1.19	42.89 ± 3.41
	4	$H1^{fx/fx} H2^{fx/fx}$	35.95 ± 2.38	7.67 ± 0.79	10.84 ± 0.86	39.96 ± 2.31

Normality calculated with Shapiro-Wilk normality test. For normal data sets, student's T-test used to calculate significance. For not normal data sets, Mann-Whitney U test used to calculate significance.

@ Data not normal # p<0.01 * p<0.05 vs H1H2control
