# Supplementary material for: Neonatal Deletion of Hand1 and Hand2 within Murine Cardiac Conduction System Reveals a Novel Role for HAND2 in Rhythm Homeostasis
Source: J Cardiovasc Dev Dis. 2022 Jul 4;9(7):214. doi: 10.3390/jcdd9070214 (PMC9324487; doi:10.3390/jcdd9070214)
Supplement: Supplementary file 1 [file jcdd-09-00214-s001.zip › Table S5.pdf]

Table S5: ECG Analysis of *Hand1LV-Cre H2*$^{fx/fx}$

Males - LEAD I

Age (weeks)	n	Genotype	Heart rate (bpm)	PR interval (ms)	QRS1 (ms)	QRS2 (ms)	QTc (ms)
5	8	*Hand1LV-Cre H2*$^{fx/fx}$	486 ± 72	31.58 ± 5.23	7.27 ± 1.51	10.78 ± 1.44	38.87 ± 5.41*
	8	*H2*$^{fx/fx}$	553 ± 54	28.68 ± 2.85	7.13 ± 0.99	10.45 ± 1.59	31.44 ± 6.42
10	7	*Hand1LV-Cre H2*$^{fx/fx}$	515 ± 32	33.72 ± 6.31$^{@*}$	7.82 ± 0.93	10.53 ± 1.15$^{@*}$	33.98 ± 6.41
	8	*H2*$^{fx/fx}$	514 ± 51	28.59 ± 1.63	7.03 ± 1.35	8.29 ± 2.69	35.54 ± 5.40
15	6	*Hand1LV-Cre H2*$^{fx/fx}$	522 ± 38	31.99 ± 2.55*	7.19 ± 1.2	9.52 ± 0.98	37.49 ± 7.98
	8	*H2*$^{fx/fx}$	546 ± 44	29.99 ± 2.55	6.51 ± 0.09	9.24 ± 1.27	40.96 ± 4.94
20	8	*Hand1LV-Cre H2*$^{fx/fx}$	536 ± 58	30.77 ± 2.65	7.76 ± 0.53	9.48 ± 0.59	36.85 ± 8.37
	8	*H2*$^{fx/fx}$	523 ± 45	29.51 ± 1.94	7.71 ± 1.04	9.97 ± 0.83	39.22 ± 7.26
25	6	*Hand1LV-Cre H2*$^{fx/fx}$	506 ± 39$^{\#}$	29.08 ± 2.37	7.84 ± 1.22	10.43 ± 1.49	37.57 ± 0.45
	7	*H2*$^{fx/fx}$	545 ± 24	28.40 ± 3.27	7.17 ± 1.15	9.69 ± 0.92	36.88 ± 0.80

Males - LEAD II

Age (weeks)	n	Genotype	PR interval (ms)	QRS1 (ms)	QRS2 (ms)	QTc (ms)
5	8	*Hand1LV-Cre H2*$^{fx/fx}$	30.35 ± 6.97	6.55 ± 0.61	9.32 ± 1.13	33.75 ± 3.90
	8	*H2*$^{fx/fx}$	29.53 ± 2.51	6.68 ± 0.61	9.32 ± 1.02	31.81 ± 6.37
10	7	*Hand1LV-Cre H2*$^{fx/fx}$	32.25 ± 5.41	6.98 ± 0.48	9.82 ± 0.85	32.74 ± 7.39
	8	*H2*$^{fx/fx}$	27.92 ± 1.58	6.68 ± 0.47	9.31 ± 0.95	38.49 ± 4.92
15	6	*Hand1LV-Cre H2*$^{fx/fx}$	31.08 ± 2.82*	6.83 ± 0.62	9.16 ± 0.52	40.03 ± 2.86
	8	*H2*$^{fx/fx}$	28.02 ± 2.80	7.23 ± 0.72	9.42 ± 0.47	34.84 ± 6.92
20	8	*Hand1LV-Cre H2*$^{fx/fx}$	31.07 ± 2.18	7.12 ± 0.69	9.60 ± 1.15	38.72 ± 5.72
	8	*H2*$^{fx/fx}$	29.68 ± 2.22	7.18 ± 0.60	9.55 ± 0.81	35.65 ± 6.62
25	6	*Hand1LV-Cre H2*$^{fx/fx}$	28.59 ± 2.86	7.48 ± 0.94	10.05 ± 1.48	36.52 ± 6.77
	7	*H2*$^{fx/fx}$	26.09 ± 6.42	7.65 ± 1.98	10.18 ± 1.92	37.34 ± 9.15

Females - LEAD I

Age (weeks)	n	Genotype	Heart rate (bpm)	PR interval (ms)	QRS1 (ms)	QRS2 (ms)	QTc (ms)
5	9	*Hand1LV-Cre H2*$^{fx/fx}$	512 ± 42	31.33 ± 1.30	6.53 ± 0.82	8.71 ± 0.93	37.05 ± 3.32
	9	*H2*$^{fx/fx}$	544 ± 39	29.40 ± 3.60	6.32 ± 0.46	9.19 ± 0.66	34.39 ± 2.48
10	9	*Hand1LV-Cre H2*$^{fx/fx}$	509 ± 34	32.21 ± 3.43	7.03 ± 0.60	8.91 ± 0.78	34.74 ± 6.11$^{\#}$
	9	*H2*$^{fx/fx}$	527 ± 50	30.67 ± 3.00	6.71 ± 0.64	9.20 ± 0.63	26.91 ± 7.37
15	9	*Hand1LV-Cre H2*$^{fx/fx}$	511 ± 53	30.89 ± 2.47	6.93 ± 0.81	9.13 ± 0.74	36.13 ± 4.03
	8	*H2*$^{fx/fx}$	527 ± 49	29.59 ± 1.92	6.90 ± 0.50	9.42 ± 0.56	36.14 ± 7.38
20	9	*Hand1LV-Cre H2*$^{fx/fx}$	495 ± 53	33.49 ± 2.98	7.47 ± 0.98	8.77 ± 0.99	37.19 ± 7.23
	8	*H2*$^{fx/fx}$	491 ± 22	31.64 ± 3.66	7.46 ± 0.58	9.52 ± 1.73	36.37 ± 5.46
25	9	*Hand1LV-Cre H2*$^{fx/fx}$	515 ± 29	34.28 ± 3.66	7.45 ± 0.55	9.31 ± 0.79	33.51 ± 4.28
	8	*H2*$^{fx/fx}$	526 ± 40	31.81 ± 3.21	7.12 ± 0.53	9.84 ± 0.65	31.49 ± 9.08

Females - LEAD II

Age (weeks)	n	Genotype	PR interval (ms)	QRS1 (ms)	QRS2 (ms)	QTc (ms)
5	9	*Hand1LV-Cre H2*$^{fx/fx}$	31.73 ± 1.43	6.51 ± 0.40	8.87 ± 0.74	31.73 ± 1.43$^{@\#}$
	9	*H2*$^{fx/fx}$	27.64 ± 3.92	6.54 ± 0.45	9.13 ± 0.68	32.91 ± 4.35
10	9	*Hand1LV-Cre H2*$^{fx/fx}$	31.86 ± 3.46	6.72 ± 0.53	9.02 ± 1.12	34.64 ± 5.39
	9	*H2*$^{fx/fx}$	31.39 ± 3.69	6.87 ± 0.53	9.12 ± 0.81	30.66 ± 7.07
15	9	*Hand1LV-Cre H2*$^{fx/fx}$	31.11 ± 3.11*	6.89 ± 0.34	8.97 ± 0.54	38.74 ± 5.97
	8	*H2*$^{fx/fx}$	28.27 ± 3.25	7.21 ± 0.45	9.45 ± 0.89	35.84 ± 7.59
20	9	*Hand1LV-Cre H2*$^{fx/fx}$	33.51 ± 3.43	7.05 ± 0.54	9.07 ± 0.95	35.00 ± 10.00
	8	*H2*$^{fx/fx}$	31.58 ± 2.30	7.58 ± 1.24	9.62 ± 0.88	34.91 ± 7.71
25	9	*Hand1LV-Cre H2*$^{fx/fx}$	32.39 ± 6.55	7.16 ± 0.73	9.33 ± 1.09	34.26 ± 4.57
	8	*H2*$^{fx/fx}$	30.49 ± 4.66	7.29 ± 0.57	9.49 ± 0.53	33.12 ± 7.96

Normality calculated with Shapiro-Wilk normality test. For normal data sets, student's T-test used to calculate significance. For not normal data sets, Mann-Whitney U test used to calculate significance.

$^{@}$ Data not normal # p<0.01 * p<0.05 vs H2control
