# Supplementary material for: Neonatal Deletion of Hand1 and Hand2 within Murine Cardiac Conduction System Reveals a Novel Role for HAND2 in Rhythm Homeostasis
Source: J Cardiovasc Dev Dis. 2022 Jul 4;9(7):214. doi: 10.3390/jcdd9070214 (PMC9324487; doi:10.3390/jcdd9070214)
Supplement: Supplementary file 1 [file jcdd-09-00214-s001.zip › Table S6.pdf]

One Way Repeated Measures Analysis of Variance
Cntn2Cre H2fxfx males

Normality Test (Shapiro-Wilk): Passed (P = 0.074)
Equal Variance Test (Brown-Forsythe): Passed (P = 0.643)

Treatment Name	N	Missing	Mean	Std Dev	SEM
5 wk	6	0	0.0298	0.00223	0.000911
10 wk	6	0	0.0286	0.0033	0.00135
15 wk	6	0	0.0257	0.00366	0.0015
20 wk	6	0	0.03	0.00601	0.00245
25 wk	6	0	0.025	0.00287	0.00117

Source of Variation	DF	SS	MS	F	P
Between Subjects	5	0.000207	0.0000413		
Between Treatments	4	0.000128	0.0000321	3.958	0.016
Residual	20	0.000162	0.0000081		
Total	29	0.000497			

All Pairwise Multiple Comparison Procedures (Student-Newman-Keuls Method) :
Comparisons for factor:

Comparison	Diff of Means	p	q	P	P<0.050
20 wk vs. 25 wk	0.00497	5	4.278	0.047	Yes
20 wk vs. 15 wk	0.00426	4	3.667	0.076	No
20 wk vs. 10 wk	0.00141	3	1.216	0.671	Do Not Test
20 wk vs. 5 wk	0.0002	2	0.172	0.904	Do Not Test
5 wk vs. 25 wk	0.00477	4	4.106	0.04	Yes
5 wk vs. 15 wk	0.00406	3	3.495	0.056	Do Not Test
5 wk vs. 10 wk	0.00121	2	1.044	0.469	Do Not Test
10 wk vs. 25 wk	0.00356	3	3.062	0.102	No
10 wk vs. 15 wk	0.00285	2	2.451	0.099	Do Not Test
15 wk vs. 25 wk	0.00071	2	0.611	0.671	Do Not Test
