# Supplementary material for: Neonatal Deletion of Hand1 and Hand2 within Murine Cardiac Conduction System Reveals a Novel Role for HAND2 in Rhythm Homeostasis
Source: J Cardiovasc Dev Dis. 2022 Jul 4;9(7):214. doi: 10.3390/jcdd9070214 (PMC9324487; doi:10.3390/jcdd9070214)
Supplement: Supplementary file 1 [file jcdd-09-00214-s001.zip › Table S7.pdf]

Table S7. Summary of epicardial voltage mapping in *H2LVCreCKO* and *H2control* hearts.

	Male		Female	
N	14	7	27	9
Genotype	*H2LVCreCKO*	*H2control*	*H2LVCreCKO*	*H2control*
LV breakthroughs<br>0<br>1<br>2<br>3	 2<br>10<br>1<br>1	 2<br>5<br>0<br>0	 2<br>23<br>2<br>0	 1<br>6<br>2<br>0
RV breakthroughs<br>0<br>1<br>2	 0<br>14<br>0	 0<br>7<br>0	 5<br>20<br>2	 2<br>7<br>0
Delay, ms	1.9 ± 1.1*	0.3 ± 0.5	1.3 ± 0.9	0.7 ± 0.8
RBBB	0	0	5	2
LBBB	2	2	2	1
Interval between RA depolarization and first epicardial breakthrough in hearts without BBB, ms	$38.3 \pm 1.2^{\#}$	29.0 ± 2.7	$40.0 \pm 1.2^{\$}$	32.8 ± 1.6

Values for delay and interval between RA depolarization and first epicardial breakthrough are mean ± SEM. *P=0.004, $^{\#}$P=0.002 and $^{\$}$P=0.009 by *t*-test versus *H2control*. RBBB, right bundle branch block; LBBB, left bundle branch block.
