# Supplementary figures and images for: Neonatal Deletion of Hand1 and Hand2 within Murine Cardiac Conduction System Reveals a Novel Role for HAND2 in Rhythm Homeostasis
Source: J Cardiovasc Dev Dis. 2022 Jul 4;9(7):214. doi: 10.3390/jcdd9070214 (PMC9324487; doi:10.3390/jcdd9070214)

**Figure S1. ECG Analysis of Cntn2Cre H1fx/fx**

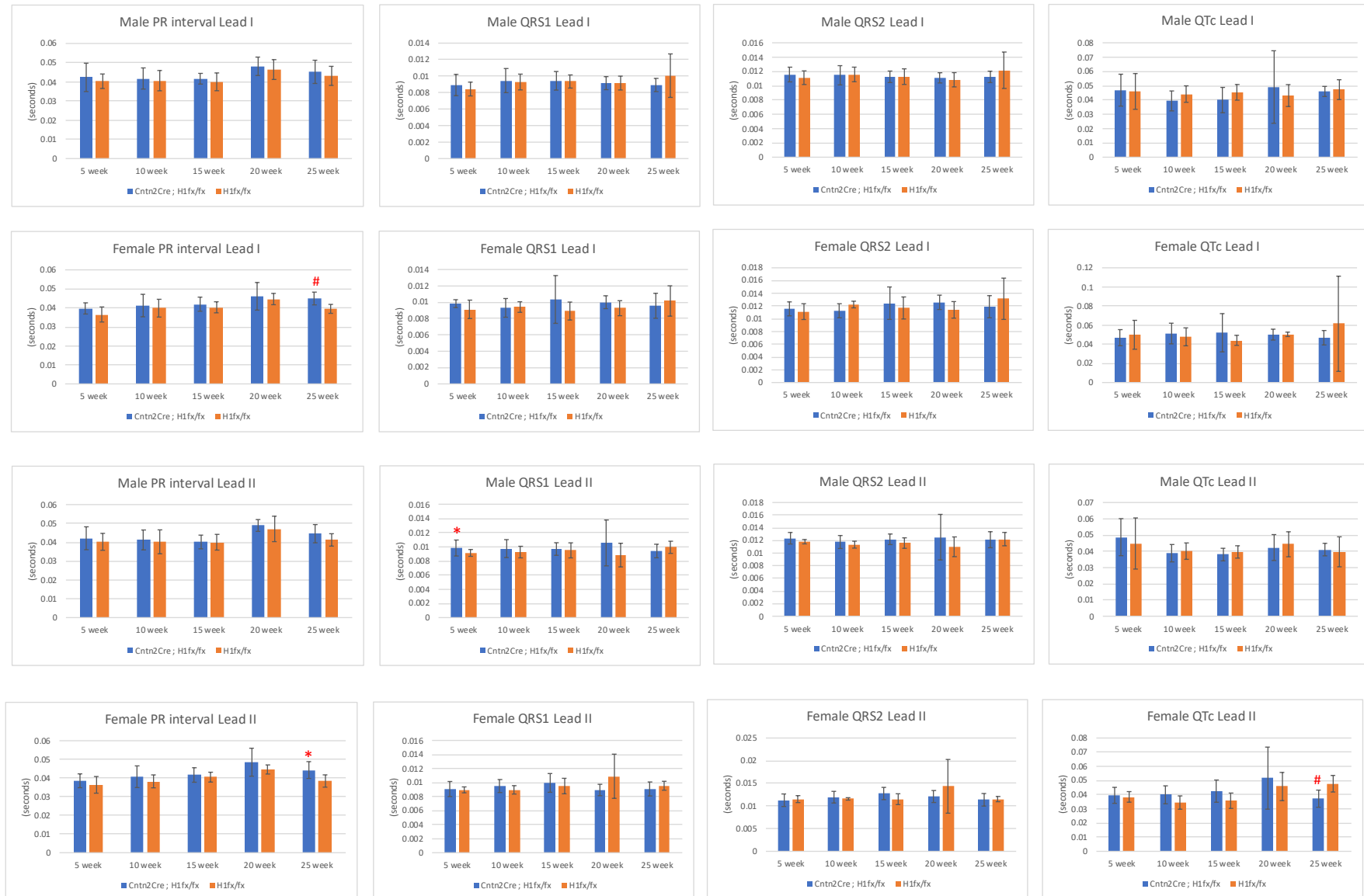

Supplement: Supplementary file 1 [file jcdd-09-00214-s001.zip › Figure S1.pdf]

**Figure S2 ECG Analysis of Cntn2Cre H2fx/fx**

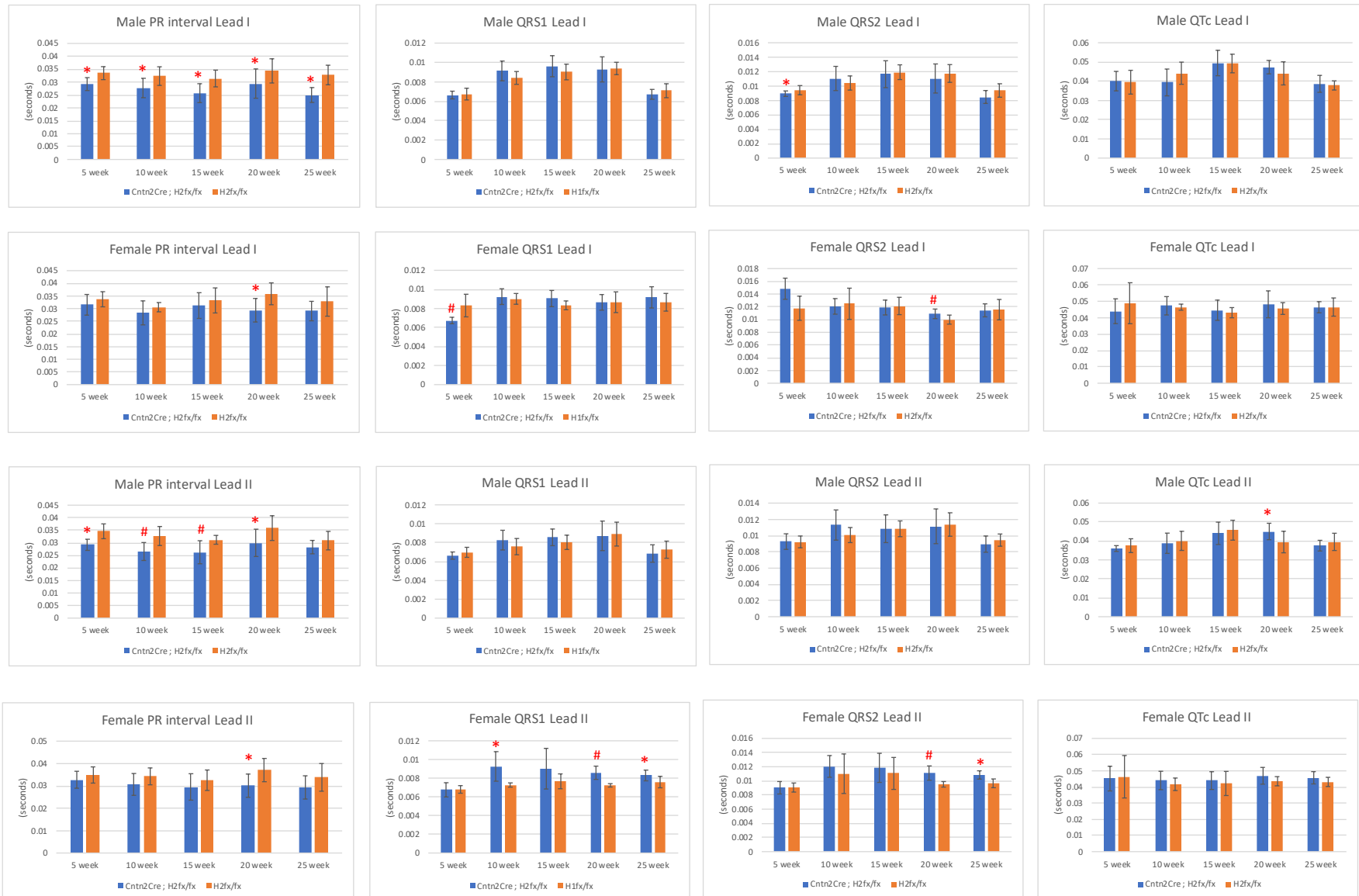

Supplement: Supplementary file 1 [file jcdd-09-00214-s001.zip › Figure S2.pdf]

**Figure S3: ECG Analysis of Cntn2Cre H1fx/fx H2fx/fx**

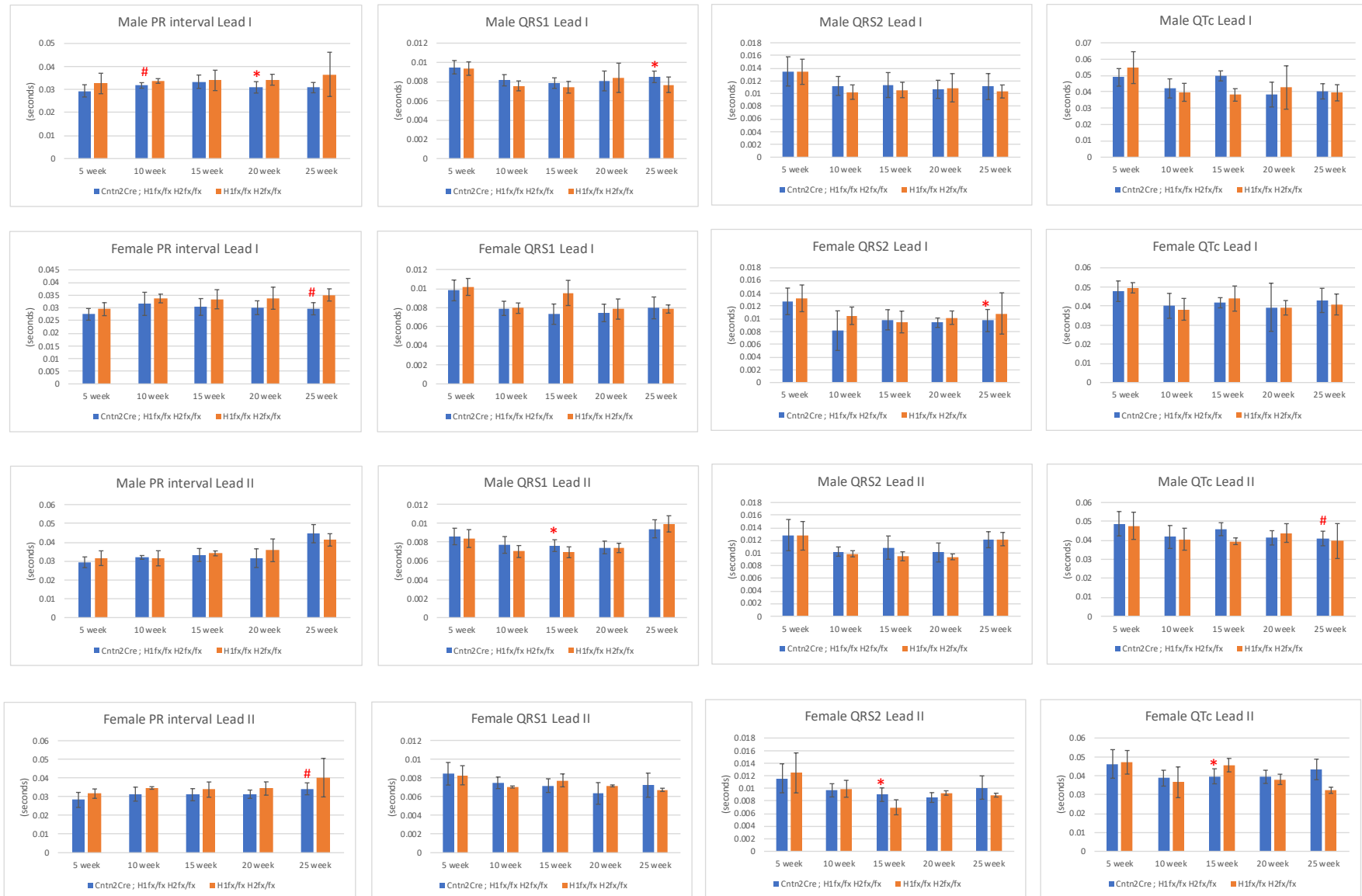

Supplement: Supplementary file 1 [file jcdd-09-00214-s001.zip › Figure S3.pdf]

**Figure S4: ECG Analysis of H1LVCre ; H2fx/fx**

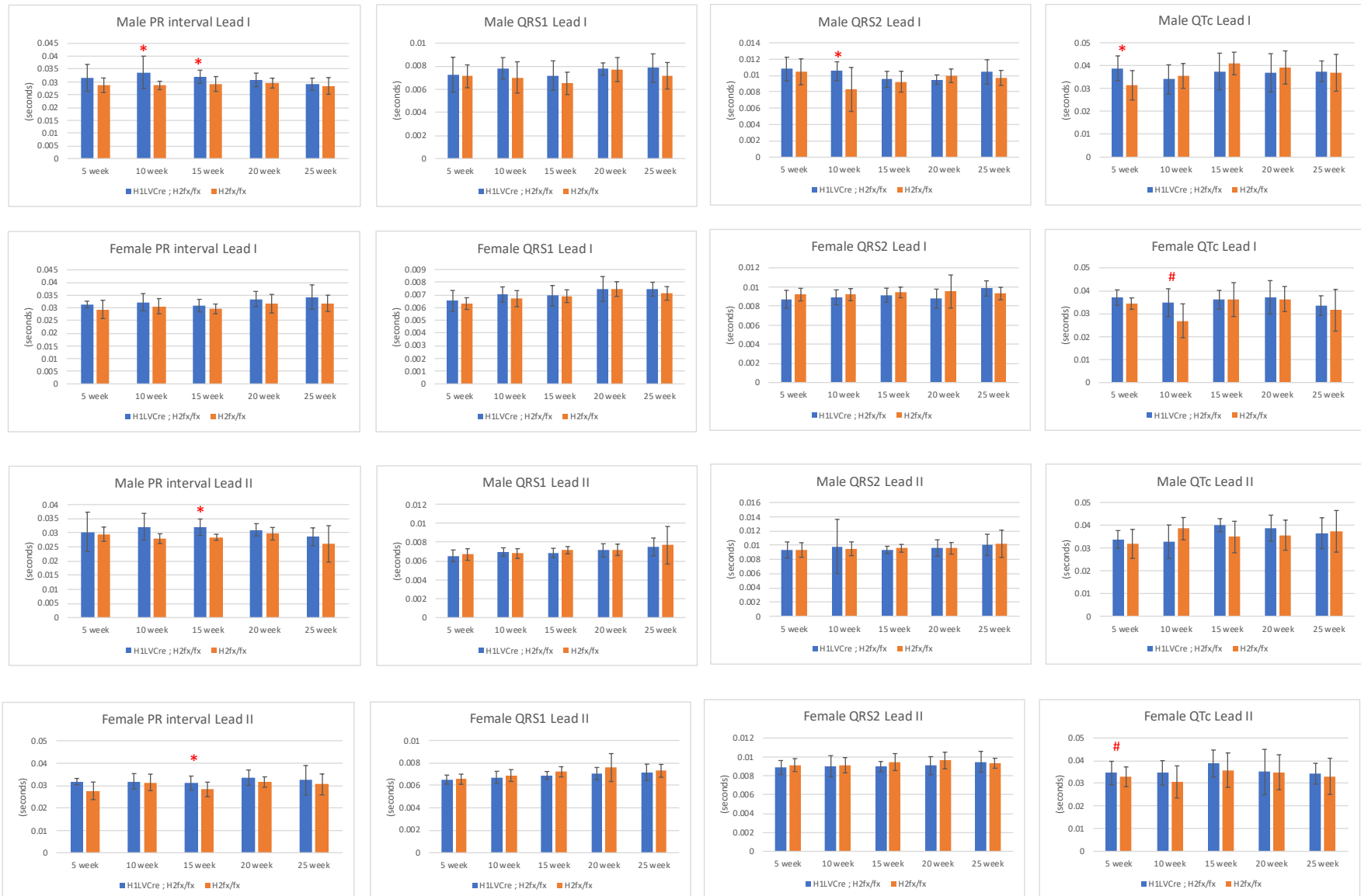

Supplement: Supplementary file 1 [file jcdd-09-00214-s001.zip › Figure S4.pdf]

Figure S5: Male vs Female control comparison

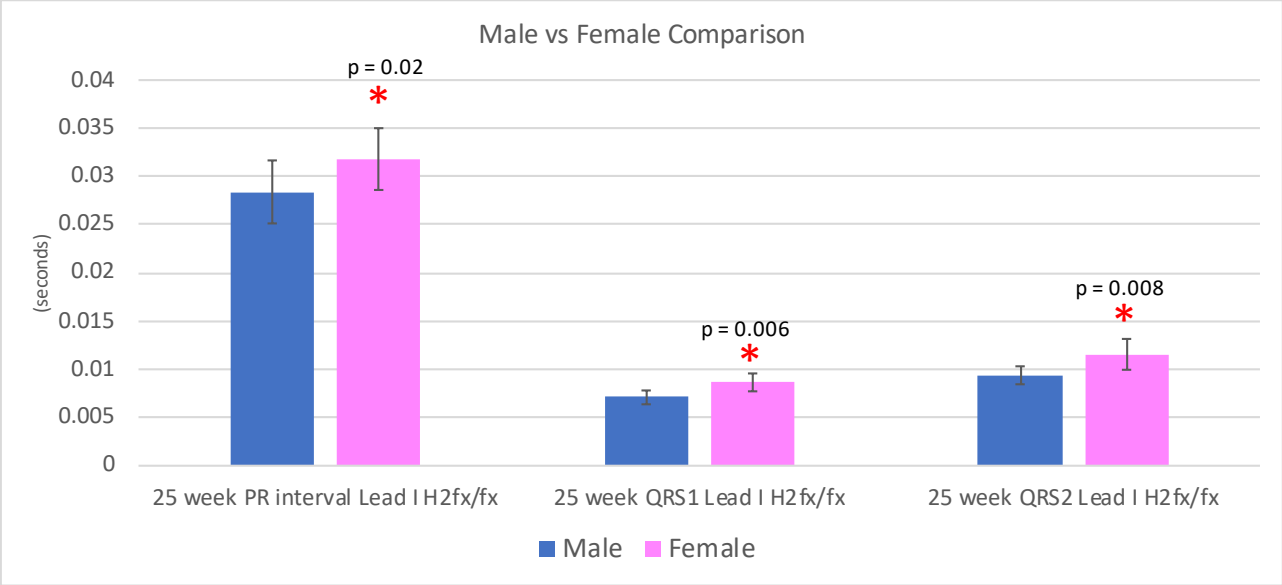

Supplement: Supplementary file 1 [file jcdd-09-00214-s001.zip › Figure S5.pdf]
